# Optimally choosing PWM motif databases and sequence scanning approaches based on ChIP-seq data

**DOI:** 10.1186/s12859-015-0573-5

**Published:** 2015-05-01

**Authors:** Michal Dabrowski, Norbert Dojer, Izabella Krystkowiak, Bozena Kaminska, Bartek Wilczynski

**Affiliations:** 1 Laboratory of Bioinformatics, Nencki Institute of Experimental Biology, Pasteura 3, Warszawa, 02-093 Poland; 2 Institute of Informatics, Univeristy of Warsaw, Banacha 2, Warszawa, 02-097 Poland; 3 Laboratory of Molecular Neurobiology, Nencki Institute of Experimental Biology, Pasteura 3, Warszawa, 02-093 Poland

**Keywords:** Transcription factor binding, Binding site, Sequence motif, Motif database

## Abstract

**Background:**

For many years now, binding preferences of Transcription Factors have been described by so called motifs, usually mathematically defined by position weight matrices or similar models, for the purpose of predicting potential binding sites. However, despite the availability of thousands of motif models in public and commercial databases, a researcher who wants to use them is left with many competing methods of identifying potential binding sites in a genome of interest and there is little published information regarding the optimality of different choices. Thanks to the availability of large number of different motif models as well as a number of experimental datasets describing actual binding of TFs in hundreds of TF-ChIP-seq pairs, we set out to perform a comprehensive analysis of this matter.

**Results:**

We focus on the task of identifying potential transcription factor binding sites in the human genome. Firstly, we provide a comprehensive comparison of the coverage and quality of models available in different databases, showing that the public databases have comparable TFs coverage and better motif performance than commercial databases. Secondly, we compare different motif scanners showing that, regardless of the database used, the tools developed by the scientific community outperform the commercial tools. Thirdly, we calculate for each motif a detection threshold optimizing the accuracy of prediction. Finally, we provide an in-depth comparison of different methods of choosing thresholds for all motifs a priori. Surprisingly, we show that selecting a common false-positive rate gives results that are the least biased by the information content of the motif and therefore most uniformly accurate.

**Conclusion:**

We provide a guide for researchers working with transcription factor motifs. It is supplemented with detailed results of the analysis and the benchmark datasets at http://bioputer.mimuw.edu.pl/papers/motifs/.

**Electronic supplementary material:**

The online version of this article (doi:10.1186/s12859-015-0573-5) contains supplementary material, which is available to authorized users.

## Background

Transcription regulation is one of the key processes that allow cells to react to environmental cues and differentiate. It would not be possible without the specific interactions between DNA-binding proteins called transcription factors (TFs) and specific target regulatory elements.

While TFs exhibit some specificity in choosing their target binding sites, this specificity is imperfect i.e. instead of binding a single specific DNA sequence a typical TF recognizes a number of similar DNA fragments with varying affinity. A pattern describing these fragments is usually called a binding *motif*, and mathematically defined with position weight matrices for almost 30 years now [1]. Importantly, the position weight matrix model makes a number of simplifying assumptions to make the model useful in practice: in particular it assumes independence between columns and additivity of the column scores. As the cost of this simplification, there comes a great advantage of being able to easily score any DNA sequence of the specified length with a log-odds measure, usually interpreted as a rough analog of the free energy of TF-DNA binding.

There has been a number of suggested extensions of the PWM model including Bayesian Markovian models [2] and so-called PBM-motifs [3] using mixtures of simpler models to alleviate the problems associated with the simplifying assumptions of the PWM model. More recently, there are also published approaches that combine the traditional PWM models with additional constraints on the physical properties of the DNA strand to be bound by a Transcription factor [4]. However, while these approaches can be useful in a few situations, it has been shown that in the vast majority of real biological cases, the benefits of the simple PWM model outweigh the potential of the more complex models to give us a slightly better description of the bindind site [5].

Given this data and the fact that PWM models are used for three decades in practically unchanged form, attests to their tremendous applicability. However, in order to use such a model to determine potential binding sites of a TF with a known motif, one needs to choose a log-odds threshold to separate between scores high enough to facilitate binding event and the non-specific sequences. Choosing such a threshold value might be guided by a natural interpretation of the log-odds score. However, already the authors of the early motif scanning tool PATSER [6] have realized that, due to the dependency of the log-odds distribution on the information content (IC) of a motif [1], the log-odds thresholds for different motifs should be different. Later, Rahmann et al. showed in [7] that there are more meaningful ways of selecting the TF binding threshold controlling for type I, type II errors or for a certain balance between them.

At the time, the number of TF motifs was limited and there were very few large scale datasets to serve as a golden standard of TF binding. Since then, the community has accumulated hundreds TF motifs in several databases [8-16] and the large-scale ChIP-seq projects such as ENCODE [17] have provided us with dozens of ChIP-seq datasets for different TFs. This wealth of data allows us now to revisit the different ways of selecting the threshold and put it in the context of different motif databases with respect to their coverage and accuracy in order to find the optimal choices for practical applications.

## Results

### Comparison of motif scanners

Comparsion of performance of motif databases should preferably be performed with the same motif scanning program, to separate the effect of the database from effects of different scanning programs. However, the two commercial motif databases: Transfac (Biobase) and MatBase (Genomatix); are each provided with a dedicated scanning program: Match [18] and MatInspector [19], respectively, using proprietary thresholds files. Therefore, as a preliminary step, we compared the performance of these dedicated programs to the performance of two motif scanners available in the public domain: matrix-scan [20] and Bio.Motif [21,22]. Both public scanners use background model-derived thresholds.

As the comparison metrics we used specificity and sensitivity, with ChIP-seq peaks for human TFs from Ensembl v.60 funcgen as the positive sets, and either third exons or genomics flanks of ChIP-seq peaks as the negative sets. The specificity and sensitivity were computed for every TF-motif pair, i.e. a pair of a TF and a related TFBS motif, and then averaged for a given database and scanning program.

Each commercial scanner was compared to the two public scanners using all motifs from its respective proprietary motif database. That is, we compared MatInspector to matrix-scan and Bio.Motif using 210 motifs from MatBase (v.8.3), representing 37 funcgen TFs; while Match was compared to the same two scanners using 106 motifs from Transfac (2010.3), representing 33 funcgen TFs. In this way, each commercial scanner could use its proprietary thresholds file. We also repeated the analysis on 32 funcgen TFs common to both motif databases, which gave nearly identical results (data not shown). For MatInspector/MatBase, in addition to individual motifs (matrices) we also used Genomatix-defined motif families (matrix families) [23].

**Figure 1:**
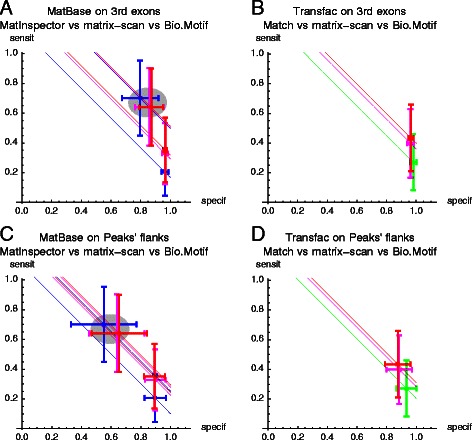
Comparison of performance of the dedicated commercial and public scanners. Shown are the average specificity and sensitivity +/−SD, for each tested database/scanner. MatIspector **(A, C)** or Match **(B, D)** were each separately compared to both matrix-scan and Bio.Motif; with either 3-rd exons **(A, B)** or flanks of the ChIP-seq peaks **(C, D)** used as the negative datasets. The color encodes scanners: matrix-scan (red), Bio.Motif (magenta), Match (green), MatInspector (blue). Stright lines through the points of average performance are the lines of equal balanced accuracy. Gray ovals in **A**, D mark the performance obtained with Genomatix motif families.

As a summary of each scanner’s performance, we present the average specificity and sensitivity over all TF-motif pairs, together with their standard deviations. These values are plotted in Figure 1. To assess trade-offs between specificity and sensitivity, we used *balanced accuracy* (BA), which is their average.

The results for individual motifs when using exons as the negative set were straightforward: the two public scanners achieved higher sensitivity then the commercial scanners, while maintaining the same uniformly high specificity (Figure 1A, B). With flanks of ChIP-seq peaks as the negative sets, the situation was similar for MatInspector used with individual motifs (Figure 1C), whereas for Match, as compared to the public scanners, there was some trade-off between specificity and sensitivity (Figure 1D), with BA higher for the two public scanners. On both types of negative sets, the use of motif families resulted in a large increase of sensitivity and balanced accuracy (Figure 1A,C), accompanied by a marked decrease of specificity.

We conclude that on individual motifs the performance of the two public scanners as measured by balanced accuracy was clearly better than of either of the two commercial scanners. When the proprietary motif families were used, MatInspectors achieved nearly the same balanced accuracy as the public scanners. However, the use of motif families resulted in a large decrease of specificity, which we consider undesirable in whole-genome applications.

For each database and type of negative set, we computed BAs for each pair of a TF and a related motif, and then we tested if BAs are significantly different between the scanners. The results (Table 1) indicated that the use of either public scanner resulted in significantly higher BA than of the respective dedicated scanner. Out of the two public scanners, we decided to use Bio.Motif for further work, because of its palette of threshold selection methods.

**Table 1:** **Significance of differences between balanced accuracies for public and commercial motif scanners**

**Motif database**	**Hypothesis tested**	**P-value for**	
		**negative dataset:**	
		**Exon 3**	**Peaks’ flanks**
Transfac	match vs matrix-scan	5.04e-41	1.13e-15
	match vs Bio.Motif	3.37e-32	1.44e-12
MatBase	MatInspector vs matrix-scan	3.98e-83	9.88e-43
	MatInspector vs Bio.Motif	3.50e-67	8.74e-36
MatBase – families	MatInspector vs matrix-scan	0.00011	0.00565
	MatInspector vs Bio.Motif	0.01066	0.03784

Entries contain p-values of the Wilcoxon rank test for the null hypothesis that the BA for the 1st scanner is not lower than the BA for the 2nd scanner.

### Comparison of databases coverage

In addition to the three long-established motif databases (Transfac, MatBase, and Jaspar), a number of new motif databases have recently been published (Table 2). These include: HOCOMOCO [14], SwissRegulon [15] and HT-SELEX [13]. The Jaspar database has recently been expanded and updated [16]. We included these new databases, alongside the current versions of the three long-established ones, into the analysis of the databases coverage, and into more in-depth, threshold independent analysis of databases performance.

**Table 2:** **Motif databases compared in the current study**

**Database**	**Number of**	**Status**	**Link**	**Publication**
	**matrices**			
	**(vertebrate)**			
HOCOMOCO v.9 (2013)	426	public	http://autosome.ru/HOCOMOCO/	[14]
Jaspar vertebrates (2014)	821	public	http://jaspar.genereg.net/	[16]
HT-SELEX (2013)	820	public	http://www.sciencedirect.com/science/ article/pii/S0092867412014961?via=ihub	[13]
SwissRegulon (2013)	190	public	http://swissregulon.unibas.ch	[15]
TRANSFAC Professional 2013.1	1435	commercial	http://www.biobase-international.com	[9]
MatBase v.9.0 (2012)	907	commercial	http://www.genomatix.de/	[23]

**Figure 2:**
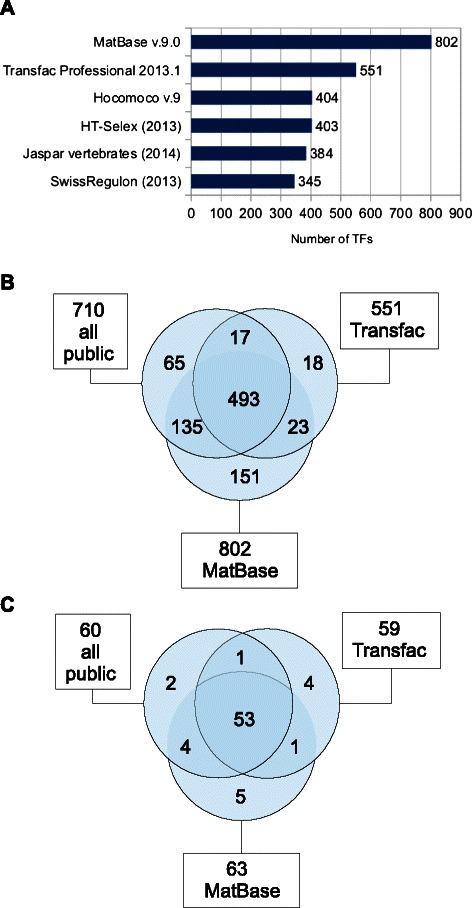
Comparsion of coverage of human TFs by motif databases.**A**. The numbers of distinct genes (Entrez Gene ID) assigned to all the vertebrate motifs from the indicated databases. For MatBase the number of TFs as provided by Genomatix is represented. **B**. The Venn diagram showing the overlap between human TF genes represented in the union of all the public databases and in the Transfac database. **C**. Similar as in **B**, but for human 81 human TFs represented in Ensembl 71 funcgen is based on MatBase v.9.0.

We first set up to compare the number of TFs represented by each database. While this is in principle a straightforward task, some care is needed due to orthology and changing gene symbols issues. Here we define the database coverage as the number of represented TFs in the human species, identified by their Entrez Gene ID. The number of distinct TFs was counted for each database (Figure 2A), for the union of all the public databases, and for the intersections between this union, Transfac, and MatBase (Figure 2B). The total number of human TFs (distinct Entrez Gene ID) assigned to the union of the public databases (710) was greater than the number of TFs assigned to the vertebrate section of Transfac Professional (551), but smaller than the number of human TF represented in the MatBase (802), with majority (493) TFs represented in each of the of the three sets (union of the public databases, Transfac, MatBase), with 65 TFs unique to the public databases, 18 TFs unique to Transfac, and 151 TFs unique to MatBase. The precise coverages change continuously between the releases, but it is notable that, at the time-point of release of the analyzed versions, the cumulative TF coverage in the public domain exceeded that of Transfac (but not MatBase), even though none of the public databases achieved this alone. In addition to the above comparisons for all human TFs, we performed a similar analysis for the subset of the 81 human TFs represented by ChIP-seq data in funcgen v.71. Out of 81 TFs represented in funcgen, 60 were represented in the union of the public databases, 59 in Transfac, and 63 in MatBase, with 53 TFs represented in each of the three sets (Figure 2C).

### Comparison of databases quality

Every motif matrix defines a log-likelihood function that discriminates between true and false binding sites. Motif scanners use matrices supplied with thresholds separating positive and negative predictions.

In order to characterize the overall quality of motif matrices, we analyzed their behavior for the whole spectrum of possible threshold values. To this aim we generated their *receiver operating characteristic* (ROC) curves and calculated *area under the curve* (AUC) for each ROC. Expected AUC value for random scoring function is 0.5 and AUC for function perfectly discriminating true and false predictions is 1.

We computed ROC and AUC for all motifs in all databases with respect to 4 datasets of negative sequences: third exons, genomic flanks of ChIP-seq peaks, random sequences with dinucleotide composition the same as in ChIP-seq peaks, and sequences generated by 3rd order Markov chains learned on peak sequences (for details see Methods section). Below we focus our analysis on AUC statistics; full results, including all ROC curves, are presented in Supplementary Materials at the authors’ website http://bioputer.mimuw.edu.pl/papers/motifs/.

**Figure 3:**
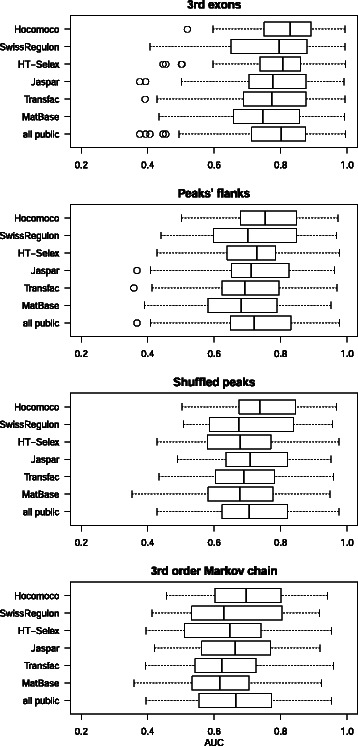
AUC distributions in motif databases. Consecutive plots present distributions of AUC calculated with respect to various negative datasets, as indicated by plots’ titles. For each motif the best related TF was selected.

Figure 3 presents AUC distributions of motif databases (for motifs having many related TFs the one that gives the highest AUC was selected). The distributions depend on the choice of the negative dataset. For example, 3rd exons are probably the least contaminated by accidental motif occurrences and yield highest AUCs (medians are around 0.8). On the other hand, the lowest AUCs are obtained for 3rd order Markov chains (medians between 0.6 and 0.7), because the high order of a Markov chain increases the chance of generating longer fragments of original sequences.

However, if one focuses on comparing AUC distributions between databases, all negative datasets yield similar picture. In all cases the highest and the lowest AUC belong to Hocomoco and MatBase motifs, respectively. The union of public databases always perform visibly better than both MatBase and Transfac. Choosing the best transcription factor for a motif favors databases having one motif for each TF over databases offering for each TF a large family of motifs. The second strategy is preferred by commercial databases (e.g. in the case of MatBase 63 TFs are represented by 377 motifs), which may to some degree explain their performance in this evaluation.

Therefore, we complement it with the analysis of AUC for 53 transcription factors represented in the funcgen ChIP-seq data and in each of the following datasets: MatBase, Transfac and the union of public databases. For each transcription factor and each database, the motif that gives the highest AUC is selected. Table 3 presents results for the negative dataset consisting of ChIP-seq peaks’ flanks (see Additional file 1 for analogous results for other negative datasets). As expected, the highest number of best motifs belongs to MatBase (19), but Jaspar has almost as many of best motifs (i.e. 16). The lowest numbers of best motifs have SwissRegulon (1) and Transfac (4). For over half of the TFs (30 out of 53) the best motif belongs to one of the public databases. AUC for motifs corresponding to the same TF are usually similar, but in some cases, for example ZBTB33, the differences are tremendous.

**Table 3:** **Comparison of optimal motifs for TFs common to MatBase, Transfac and the union of public databases**

**ENCODE/funcgen**	**MatBase**		**Transfac Prof.**		**Jaspar vert.**		**Hocomoco**		**HT-Selex**		**SwissRegulon**	
**TF name**	**AUC**	**matrix name**	**AUC**	**matrix name**	**AUC**	**matrix name**	**AUC**	**matrix name**	**AUC**	**matrix name**	**AUC**	**matrix name**
ATF3	**0.710**	**V$CREB.02**	0.693	M00981	–	–	0.694	M00015	–	–	–	–
Ap2alpha	0.809	V$AP2.02	0.807	M01045	**0.816**	**MA0003.2**	0.777	M00004	0.779	selex292	0.754	TFAP2A,C.p2
Ap2gamma	**0.799**	**V$AP2.02**	0.765	M00470	0.792	MA0524.1	0.781	M00006	0.784	selex298	0.751	TFAP2A,C.p2
BHLHE40	0.947	V$BHLHB2.01	0.799	M01034	0.885	MA0464.1	0.955	M00022	**0.958**	**selex316**	0.917	ARNT_ARNT2_BHLHB2_
												MAX_MYC_USF1.p2
CTCF	0.929	V$CTCF.04	0.931	M01259	**0.942**	**MA0139.1**	0.940	M00045	0.922	selex2	0.934	CTCF.p2
Cfos	0.751	V$AP1.01	0.759	M00517	0.744	MA0476.1	**0.760**	**M00093**	–	–	0.742	FOS_FOSB,L1_JUNB,D.p2
Cjun	0.814	V$AP1.01	0.820	M00925	0.799	MA0099.1	**0.827**	**M00183**	–	–	0.570	JUN.p2
Cmyc	0.700	V$CMYC.01	0.685	M00322	**0.702**	**MA0147.1**	0.690	M00216	–	–	0.659	ARNT_ARNT2_BHLHB2_
												MAX_MYC_USF1.p2
E2F1	**0.802**	**V$E2F3.01**	0.764	M00938	0.753	MA0024.2	0.694	M00052	0.723	selex750	0.674	E2F1..5.p2
E2F4	0.682	V$E2F4.01	0.678	M00920	**0.689**	**MA0470.1**	0.650	M00055	0.502	selex753	0.654	E2F1..5.p2
E2F6	**0.698**	**V$E2F4.01**	0.435	M01252	0.653	MA0471.1	0.681	M00057	–	–	–	–
EBF	0.740	V$EBF1.01	0.736	M01871	0.721	MA0154.2	0.746	M00037	**0.751**	**selex79**	0.692	EBF1.p2
ELF1	**0.862**	**V$ELK1.03**	0.835	M02053	0.800	MA0473.1	0.832	M00065	0.836	selex81	0.797	ELF1,2,4.p2
ETS1	**0.765**	**V$ELK3.01**	0.753	M02063	0.680	MA0098.1	0.708	M00082	0.765	selex100	0.683	ETS1,2.p2
Egr1	0.831	V$EGR1.01	0.848	M01972	0.822	PB0010.1	0.844	M00060	**0.853**	**selex3**	0.841	EGR1..3.p2
FOSL1	0.885	V$AP1.02	0.890	M00517	**0.890**	**MA0477.1**	0.865	M00091	–	–	0.881	FOS_FOSB,L1_JUNB,D.p2
FOSL2	0.877	V$AP1.01	0.870	M00925	0.865	MA0478.1	**0.885**	**M00092**	–	–	0.852	FOSL2.p2
FOXA1	0.763	V$FREAC4.01	0.826	M01261	**0.832**	**MA0148.3**	0.808	M00094	–	–	–	–
FOXA2	0.759	V$FREAC4.01	0.735	M02014	**0.834**	**MA0047.2**	0.816	M00095	–	–	**0.834**	**FOXA2.p3**
Gabp	0.873	V$ELK1.03	0.867	M02074	**0.879**	**MA0062.2**	0.876	M00116	0.871	selex116	0.870	ELK1,4_GABPA,B1.p3
Gata1	**0.711**	**V$GATA5.01**	0.685	M00203	0.683	MA0035.3	0.697	M00117	–	–	0.479	GATA1..3.p2
Gata2	**0.853**	**V$GATA2.03**	0.834	M00789	0.834	MA0036.2	0.843	M00118	–	–	0.538	GATA1..3.p2
HNF4A	0.801	V$HNF4.01	0.838	M02220	0.847	MA0114.2	**0.850**	**M00147**	0.837	selex673	0.809	HNF4A_NR2F1,2.p2
HNF4G	0.864	V$HNF4.01	0.811	M00764	**0.898**	**MA0484.1**	0.788	M00148	–	–	–	–
IRF4	**0.669**	**V$ISRE.01**	0.641	M00772	0.648	PB0034.1	0.603	M00174	0.665	selex148	–	–
Junb	0.912	V$AP1.01	0.912	M00925	**0.920**	**MA0490.1**	0.911	M00181	–	–	0.900	FOS_FOSB,L1_JUNB,D.p2
Jund	0.820	V$AP1.01	0.823	M00925	0.817	MA0491.1	**0.827**	**M00182**	–	–	0.805	FOS_FOSB,L1_JUNB,D.p2
MEF2A	0.643	V$MEF2.02	0.650	M00231	**0.653**	**MA0052.2**	0.616	M00204	0.615	selex156	0.604	MEF2A,B,C,D.p2
MEF2C	**0.721**	**V$MEF2.02**	0.682	M00941	0.719	MA0497.1	0.664	M00205	–	–	0.669	MEF2A,B,C,D.p2
Max	**0.738**	**V$CMYC.01**	0.703	M00322	0.700	PB0043.1	0.720	M00199	0.730	selex326	0.711	ARNT_ARNT2_BHLHB2_
												MAX_MYC_USF1.p2
NFKB	**0.896**	**V$NFKAPPAB65.02**	0.891	M00774	0.878	MA0105.3	0.872	M00235	0.776	selex189	0.861	NFKB1_REL_RELA.p2
NR4A1	0.512	V$NBRE.01	0.492	M01217	–	–	**0.542**	**M00259**	–	–	–	–
Nanog	0.560	V$HOXA2.01	**0.631**	**M01247**	–	–	0.556	M00221	–	–	0.630	NANOGmouse.p2
Nfe2	0.855	V$NFE2.01	0.846	M00037	0.877	MA0501.1	**0.882**	**M00231**	0.771	selex392	0.835	NFE2.p2
Nrf1	0.951	V$NRF1.01	0.969	M00652	0.963	MA0506.1	0.973	M00264	**0.977**	**selex194**	0.968	NRF1.p2
Nrsf	0.838	V$NRSF.02	**0.879**	**M01256**	0.850	MA0138.2	0.854	M00316	–	–	0.847	REST.p3
POU2F2	**0.513**	**V$OCT1.02**	0.498	M00210	0.481	MA0507.1	0.504	M00290	0.503	selex232	0.503	POU2F1..3.p2
POU5F1	0.868	V$OCT3_4.02	0.857	M01125	**0.881**	**MA0142.1**	0.874	M00294	–	–	0.857	POU5F1_SOX2dimer.p2
PU1	**0.932**	**V$SPI1.05**	0.884	M01203	0.914	MA0080.3	0.922	M00350	0.860	selex123	0.884	SPI1.p2
Pax5	0.613	V$PAX5.01	0.613	M00143	0.713	MA0014.2	0.729	M00274	**0.768**	**selex200**	0.606	PAX5.p2
Pbx3	0.739	V$PBX1_MEIS1.01	0.546	M00998	–	–	**0.758**	**M00280**	–	–	–	–
RXRA	0.714	V$PPARG.03	0.608	M02272	0.707	MA0065.1	0.693	M00326	**0.731**	**selex710**	0.720	RXRG_dimer.p3
SP1	0.559	V$SP1.03	0.552	M00932	**0.561**	**MA0079.3**	0.555	M00346	0.547	selex29	0.551	SP1.p2
SP2	0.711	V$SP4.01	0.719	M01783	**0.726**	**MA0516.1**	0.676	M00347	–	–	–	–
Srf	0.681	V$SRF.05	**0.693**	**M00186**	0.661	MA0083.1	0.657	M00355	0.657	selex159	0.656	SRF.p3
Tcf12	**0.723**	**V$ASCL2.01**	0.679	M00698	0.712	MA0521.1	0.703	M00152	–	–	0.574	TAL1_TCF3,4,12.p2
Tr4	0.601	V$HNF4.01	0.644	M01776	**0.652**	**MA0504.1**	0.623	M00256	0.611	selex676	–	–
USF1	**0.947**	**V$USF1.02**	0.936	M00121	0.903	MA0093.2	0.945	M00396	0.935	selex352	0.932	ARNT_ARNT2_BHLHB2_
												MAX_MYC_USF1.p2
Yy1	**0.778**	**V$YY1.03**	0.723	M02044	0.713	MA0095.2	0.735	M00394	0.756	selex33	0.657	YY1.p2
ZBTB33	0.489	V$KAISO.01	0.517	M01119	**0.881**	**MA0527.1**	0.749	M00184	–	–	–	–
ZBTB7A	**0.699**	**V$ZF9.01**	0.682	M01100	–	–	0.640	M00404	0.632	selex37	–	–
ZEB1	**0.766**	**V$ZEB1.01**	0.689	M00414	0.752	MA0103.2	0.686	M00409	–	–	0.734	ZEB1.p2
Znf263	0.685	V$ZNF263.01	**0.762**	**M01587**	0.653	MA0528.1	–	–	–	–	–	–

For each TF the motif with the highest AUC from each database is presented. The best motifs from all databases and the corresponding AUC are bolded (note that for FOXA2 motifs from Jaspar and SwissRegulon are both optimal). AUC are calculated with respect to negative sequences composed of flanks of ChIP-seq peaks.

### Selection of log-odds thresholds

As was mentioned previously, motif matrices define log-likelihood functions that should be supplied with thresholds separating positive and negative predictions. Selection of such a threshold determines the balance between prediction sensitivity and specificity. Usually, a reasonable solution is to maximize balanced accuracy, i.e. the mean of sensitivity and specificity.

Therefore we calculated maximal balanced accuracies and corresponding thresholds for all transcription factors represented in our benchmark dataset and related motifs. Results are presented in Additional file 2.

We also decided to examine generic threshold selection approaches, i.e. methods setting the threshold on the basis the motif matrix only. They have considerable advantages – simplicity (calculations are much easier) and wide applicability (no benchmark dataset is required).

Probably the simplest generic approach is to set a common log-likelihood threshold for all motifs. Unfortunately, log-likelihood distributions substantially vary across motifs and the same threshold value may result in underestimating occurrences for one motif and overestimating them for the other. Therefore several score distribution based approaches for threshold selection were proposed. In the current study we analyze 3 representative methods for threshold selection implemented in the Bio.Motif package: FPR, FNR and balanced (see 3 for details).

It should be noted that all these approaches (including pure log-likelihood score) are parameterized and in each case the parameter enables the user to select any sensitivity-specificity configuration obtainable for a given motif. In particular, each method allows optimizing balanced accuracy for individual motifs. Therefore the point in which the approaches differ lies in the ability to select the threshold consistently across motifs.

**Figure 4:**
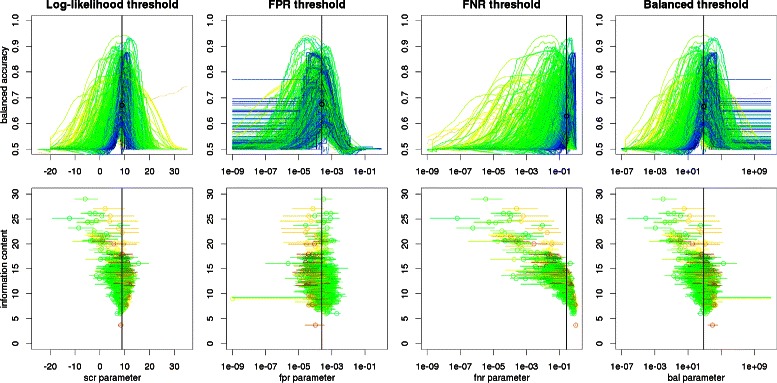
Balanced accuracies for various approaches to threshold selection. Top row: balanced accuracy vs threshold parameter. Colors represent motif information content: from blue (low), through green and yellow to beige (high). Vertical black lines indicate optimal thresholds, black circles indicate corresponding average balanced accuracies. Bottom row shows how (sub-)optimal parameter values of a motif (X-axis) depends on its information content. For each motif, a circle represents parameter value yielding maximal balanced accuracy and a horizontal line represents a parameter range, for which BA is at least 95*%* of the maximum. Colors represent motif AUC: from green (low), through yellow to red (high). Balanced accuracies are calculated with respect to negative sequences composed of flanks of ChIP-seq peaks.

**Figure 5:**
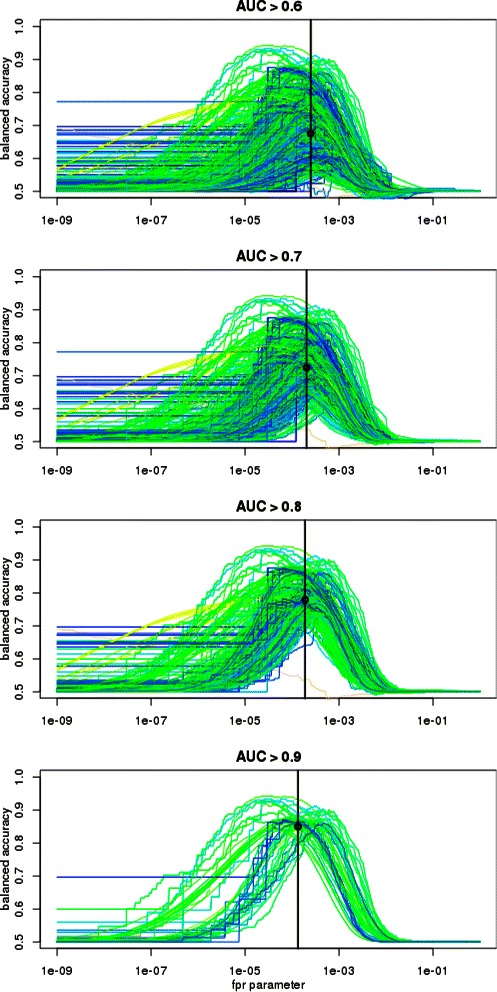
Balanced accuracy versus the FPR threshold for various AUC > 0.6, AUC > 0.7, AUC > 0.8, AUC > 0.9, cutoffs. Colors etc. as on Figure 4, top row.

Figure 4 presents the relation between balanced accuracy (calculated with respect to negative sequences composed of flanks of ChIP-seq peaks; for other negative datasets see Additional file 1)

and the parameter of the selection method for all motifs in our benchmarking dataset. In order to avoid noise introduced by poor motifs, we restricted our attention to matrices having AUC>0.6. Extreme parameters yield extreme values of sensitivity and specificity (one equal to 0 and the other to 1), resulting in the balanced accuracy equal to 0.5. Therefore BA is maximized for intermediate parameters for every motif. The parameter values giving the maximum of average BA across all motifs is indicated by vertical black lines and listed in Table 4. The highest average BA is obtained with FPR approach, since its BA profiles are most consistent – the bulk of motifs gain near-maximal BA for *α*∈[10^−4^;10^−3^](see 3 section for the definition of *α*). This is even more evident when motifs with lower AUC are excluded (see Figure 5). On the other hand, for the rest of approaches the location of BA peak seems to be strongly correlated with the information content (IC) of a motif (see Figure 4, bottom). This observation suggests that the prediction accuracy may be improved by using threshold parameters dependent on IC and other motif features (such method still might be used for any motif, without the need of a benchmark dataset).

**Table 4:** **Average balanced accuracy for optimal threshold parameters**

**Threshold selection**	**Average BA for parameter:**		**Optimal constant parameter for negative dataset:**				
**method**							
	**Optimal constant**	**Fitted with linear model**		**Peaks’ flanks**	**3rd exons**	**Shuffled peaks**	**3rd order MCs**
Log-likelihood	0.6704	0.6724		8.9	8	9	9.9
FPR	0.6749	0.6757		0.00025	0.00041	0.00021	0.00013
FNR	0.6285	0.6641		0.26	0.18	0.27	0.32
Balanced	0.6664	0.6743		794	457	977	2089

Only motifs with AUC>0.6 are considered. Balanced accuracies are calculated with respect to negative sequences composed of flanks of ChIP-seq peaks. 2nd column contains average balanced accuracies for thresholds in 4th column. 3rd column contains average balanced accuracies for thresholds linearly depending on the motif features: information content, log-likelihood value range, log-likelihood standard deviation.

Therefore we selected several characteristics of the log-likelihood distribution under the background model (i.e. the theoretical distribution of the log-likelihood function of sequences randomly generated from the background model). Namely, we considered expected value (which equals to the motif IC), standard deviation, minimum and maximum. We fitted a linear regression model describing optimal threshold parameters by these characteristics.

The averages of BA (calculated with respect to negative sequences composed of flanks of ChIP-seq peaks) for constant parameters and parameters predicted from motif features are compared in Table 4. As expected, linear model visibly improved average BAs for methods with worse result for constant parameters, narrowing the range of average BAs to interval [0.6641;0.6757]. However, the average BA 0.6749 for constant FPR threshold was not reached by any of the other methods. To have the proper perspective to assess these differences, the reader should be aware of the distance between these values and the upper bound of the average BA for considered motifs. The ideal method would select for each motif a threshold yielding its optimal balanced accuracy. Consequently, it would reach a result equal to the average of BA maxima, which is 0.6949 for our dataset. Thus setting individual thresholds for each motif may improve the average BA by 0.02, when compared to thresholds globally set with FPR parameter *α*=0.0003.

Estimated optimal parameters obviously depend on the choice of the negative dataset (see the last 4 columns of Table 4), but the dispersions are relatively small: the log-likelihood range is less 2 and the ratios between the extremes for other parameters (the logarithmic scale is more suitable here) are ∼3. Moreover, the variability of optimal parameters is consistently explained by the characteristics of negative datasets - the lower are AUC for a dataset, the sharper is the parameter.

## Discussion and conclusion

We have aimed to analyze the coverage and accuracy of different motif databases as well as the optimality of different approaches to motif scanning.

As one of the key advantages of the public resources is the possibility of freely combining them, we included into our analysis as an important category the union of the public databases.

In respect of the coverage of human TFs the two commercial databases maintain their lead as compared to each of the public databases separately (Figure 2A). However, the union of the public databases achieves coverage comparable to that of the commercial databases (Figure 2B).

In respect of motifs’ performance as judged by the distributions of the AUC, all the compared databases produce broadly similar results, with the median AUC higher for the union of the public databases than for either commercial one (Figure 3). At the single database level, Hocomoco was consistently the best performer for all choices of the negative datasets. Relative performance of some of the databases, in particular Jaspar and SwissRegulon, depended on the choice of the negative dataset. Jaspar performed better on peaks’ flanks and negative datasets generated from Markov models, while SwissRegulon performed better on third exons.

As another measure of motifs’ performance, we identified which database supplied the best-performing motif (Table 3) for every TF represented across all the databases. In more than half of the cases, the best-performing motif originated from the public databases. At the single database level, the largest number of best motifs was supplied by MatBase, but with Jaspar and Hocomoco at the second and third position supplying together more best motifs than MatBase.

In conclusion, public databases together match the coverage and the quality of their commercial counterparts, with no cost and limits of use imposed by the latter. On the other hand, an overhead associated with combining several public resources (unifying formats, installing updates, etc.) and differences in content other than the PWMs, make the choice public vs commercial not always obvious.

Given a sizeable number of TF motifs available only in one of the databases, a researcher going to predict binding of a particular TF needs a guide that helps to find the most appropriate public database. Our findings provide such a guide for a number of human TFs supported by reliable experimental data. Moreover, the usability of such guides may increase in a near future, when one can expect that the contents of several public databases will be available from a single server.

In terms of the performance of different scanning tools, publicly available mature software packages have exceeded the commercial tools in accuracy. While this might be at least in part a result of the commercial providers reluctance to change the behavior of the tools their customers depend on, we would advise researchers to use the publicly available tools.

With respect to the choice of the optimal threshold for multiple motifs, the method based on controlling the false positive rate is clearly the one least biased by the information content of the motif and therefore the most consistent between motifs.

In conclusion, the recent increase in availability of both sequence motifs and binding data have given us the opportunity to assess different motif databases and scanning methods for predicting potential TF binding sites. The results allow us to give some particular recommendations (such as the choice of the best motif and corresponding optimal threshold for a given TF) as well as general conclusions (superiority of FPR measure and public scanners) for users of these databases. We believe that our findings will prove to be useful also for hybrid methods [24,25], which use other data to improve motif-based predictions of binding sites.

## Methods

### Parsing and annotation of motif databases

Database distribution files downloaded from their providers websites (Table 1) were parsed using custom-made scripts to yield matrices in the common format, as well as matrices’ annotation to transcription factors. For the public databases, the available identifiers were the TF gene symbols or UniProt identifiers. For the purpose of comparison and mapping to the ChIP-seq datasets, these identifiers were mapped to human Entrez Gene ID, using the db2db function of the bioDBNet webservice [26] http://biodbnet.abcc.ncifcrf.gov/webServices/bioDBnet.wsdl. For the commercial databases the Entrez gene IDs were directly available. Genomatix uses the concept of matrix family [23], here referred to as ‘motif family’, i.e. family of the motifs so similar that they are predicted to bind a common set of TF orthologs in related species (e.g. all vertebrates). In Additional file 3 we provide a comprehensive mapping table for each motif database, for the commercial databases limited to the motifs used in this work.

### Preparation of positive and negative TF-binding datasets

The positive and the negative sequences were obtained from the Nencki Genomics Database - NGD [27] http://www.nencki-genomics.org and were based on Ensembl [28] funcgen. For the comparison of the scanner performance we used TF ChIP-seq data for 44 TFs + CTCF from funcgen v.60 as the positive set. For the comparison of databases performance we used ChIP-seq data for 80 TFs + CTCF from funcgen v.71 as the positive set. We considered 4 negative datasets:

- all human third exons, excluding exons with UTRs (common for all TFs),
- sequences flanking ChIP-seq peaks for particular TFs (each peak shifted by its length + 40 nt along the chromosome),
- random sequences of length and dinucleotide composition following ChIP-seq peaks for particular TFs, generated by BiasAway [29],
- random sequences of length following ChIP-seq peaks for particular TFs, generated by 3rd order Markov chains learned on these peaks.

All sequences are available in Supplementary Materials at the authors’ website http://bioputer.mimuw.edu.pl/papers/motifs/.

The following quality measures were used in the comparison:

- *Sensitivity* – the proportion of ChIP-seq peaks that contain at least one predicted binding site,
- *Specificity* – the proportion of fragments in the negative dataset that contain no predicted binding site,
- *Balanced accuracy* – the average of sensitivity and specificity.

### Mapping of TFs to gene identifiers

Some TFs were represented by more than one ChIP-seq dataset and many TFs were mapped to several motifs from each database (for the details see Supplementary Materials). The annotation of the TF ChIP-seq datasets in Ensembl funcgen v.71 was downloaded from the Ensembl website (http://apr2013.archive.ensembl.org/Homo_sapiens/Experiment?db=core;ex=project-ENCODE-). The Ensembl gene IDs of the features in the classes: ‘Transcription factor’ and ‘Insulator’ (for CTCF) were mapped to Entrez Gene ID using the aforementioned db2db function of the bioDBNet webservice. For one entry (ZEB1), its Ensembl gene ID was corrected (to ENSG00000148516) prior to the mapping.

### Motif scanning

The two commercial motif databases: Transfac Professional (Biobase) and MatBase (Genomatix) are distributed with dedicated motif scanning programs: Match [18] and MatInspector [19,30], respectively, and proprietary thresholds files, aimed at controlling the false positive rate. For matrix-scan we choose 1st order Markov chain background model learned on human gene upstream no-orf sequences and for Bio.Motif we used the uniform background model. We choose 0.0001 chance of Type I error as the threshold for both public scanners, because it yielded similar numbers of genome-wide matches to Match or MatInspector when run with the two respective motif databases.

### Computation of intersections

For the comparison of scanners, we computed genome-wide intersections between ChIP-seq peaks and motifs using stored procedures of the NGD database [27] http://www.nencki-genomics.org. The NGD database stores the TFs ChIP-seq data imported from funcgen and the results of genome-wide motif scanning. The intersections, specificity and sensitivity were computed separately for each dataset-motif pair, then averaged twice: first for each TF-motif pair, then for a given scanner/database. For the in-depth comparison of performance we used an extended set of motif databases: Jaspar (2014) vertebrates, Transfac Professional 2013.1, MatBase 9.0, HOCOMOCO v.9, HT-SELEX, SwissRegulon (2013). This time we scanned only the positive and the negative sequences with the Bio.Motif scanner. As the average length of human third exons (153 +/− 304) was smaller than the average size of the ChIP-seq peaks (403 +/− 172), before the scanning we added 100-nt flanks of either side of exons 3 and use these flanked exons as the negative set.

### Threshold selection methods

Given a motif matrix *M*, the log-likelihood of a sequence *w* of corresponding length is given by

$$L_{M}(w)=\log\frac{P(w|M)}{P(w|B)} $$

where *P*(*w*|*M*) is the probability of observing *w* given the motif model and *P*(*w*|*B*) is the probability of observing *w* given the background model. Given a threshold *t*_*M*_, all sequences *w* satisfying *L*_*M*_(*w*)>*t*_*M*_ are classified as *M*-occurrences.

Since log-likelihood distributions substantially vary across motifs, there were proposed approaches for threshold selection based on the shape of these distributions. Some representative methods are implemented in the Bio.Motif package:

- *FPR* approach aims at restricting the number of false positive motif occurrences. For assumed type I error level *α*, *t*_*M*_ is chosen to satisfy *P*(*L*_*M*_(*w*)>*t*_*M*_|*B*)=*α*.
- *FNR* approach restricts the number of false negatives. In this method *t*_*M*_ satisfies *P*(*L*_*M*_(*w*)<*t*_*M*_|*M*)=*β* for assumed type II error level *β*.
- *Balanced* approach constrains the proportion between the levels of both errors, i.e. threshold *t*_*M*_ satisfies *P*(*L*_*M*_(*w*)<*t*_*M*_|*M*)=*γ*·*P*(*L*_*M*_(*w*)>*t*_*M*_|*B*) for assumed parameter *γ*. Setting *γ* to the inverse of the expected frequency of motif occurrences results in roughly the same number of false positive and false negative binding site predictions.

## Additional files

Additional file 1
**Supplementary tables and figures with results for alternative negative datasets.**

Additional file 2
**Performance summary for all TF-motif pairs and all negative datasets.**

Additional file 3
**TF-motif mappings for all databases.**
