# Supplementary material for: Optimally choosing PWM motif databases and sequence scanning approaches based on ChIP-seq data
Source: BMC Bioinformatics. 2015 May 1;16:140. doi: 10.1186/s12859-015-0573-5 (PMC4436866; doi:10.1186/s12859-015-0573-5)
Supplement: Additional file 1 — Supplementary tables and figures with results for alternative negative datasets. [file 12859_2015_573_MOESM1_ESM.pdf]

Optimally choosing PWM motif databases and sequence scanning approaches based on ChIP-seq data – Supplementary materials

Michal Dabrowski, Norbert Dojer, Izabella Krystkowiak, Bozena Kaminska, Bartek Wilczynski

List of Figures

1 Balanced accuracies for various approaches to threshold selection (vs 3rd exons). 2
2 Balanced accuracies for various approaches to threshold selection (vs shuffled peaks). 3
3 Balanced accuracies for various approaches to threshold selection (vs 2nd order Markov chain). . 4
4 Balanced accuracies for various approaches to threshold selection (vs 3rd order Markov chain). . 5
5 Balanced accuracy versus the FPR threshold for various AUC cutoffs (vs 3rd exons). 6
6 Balanced accuracy versus the FPR threshold for various AUC cutoffs (vs shuffled peaks). 7
7 Balanced accuracy versus the FPR threshold for various AUC cutoffs (vs 2nd order Markov chain). 8
8 Balanced accuracy versus the FPR threshold for various AUC cutoffs (vs 3rd order Markov chain). 9

List of Tables

1 Optimal motifs for negative dataset **3rd exons** 10
2 Optimal motifs for negative dataset **Shuffled peaks** 11
3 Optimal motifs for negative dataset **2nd order Markov chain** 12
4 Optimal motifs for negative dataset **3rd order Markov chain** 13

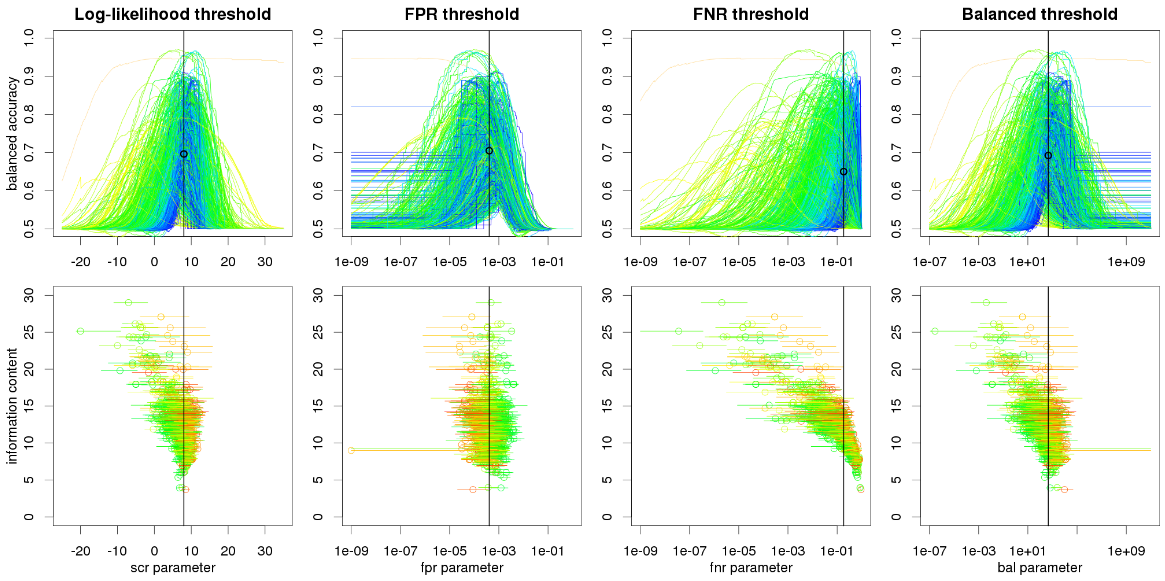

Figure 1: Balanced accuracies for various approaches to threshold selection (vs 3rd exons). Top row: balanced accuracy vs threshold parameter. Colors represent motif information content: from blue (low), through green and yellow to beige (high). Vertical black lines indicate optimal thresholds, black circles indicate corresponding average balanced accuracies. Bottom row shows how (sub-)optimal parameter values of a motif (X-axis) depends on its information content. For each motif, a circle represents parameter value yielding maximal balanced accuracy and a horizontal line represents a parameter range, for which BA is at least 95% of the maximum. Colors represent motif AUC: from green (low), through yellow to red (high). Balanced accuracies are calculated with respect to negative sequences composed of 3rd exons.

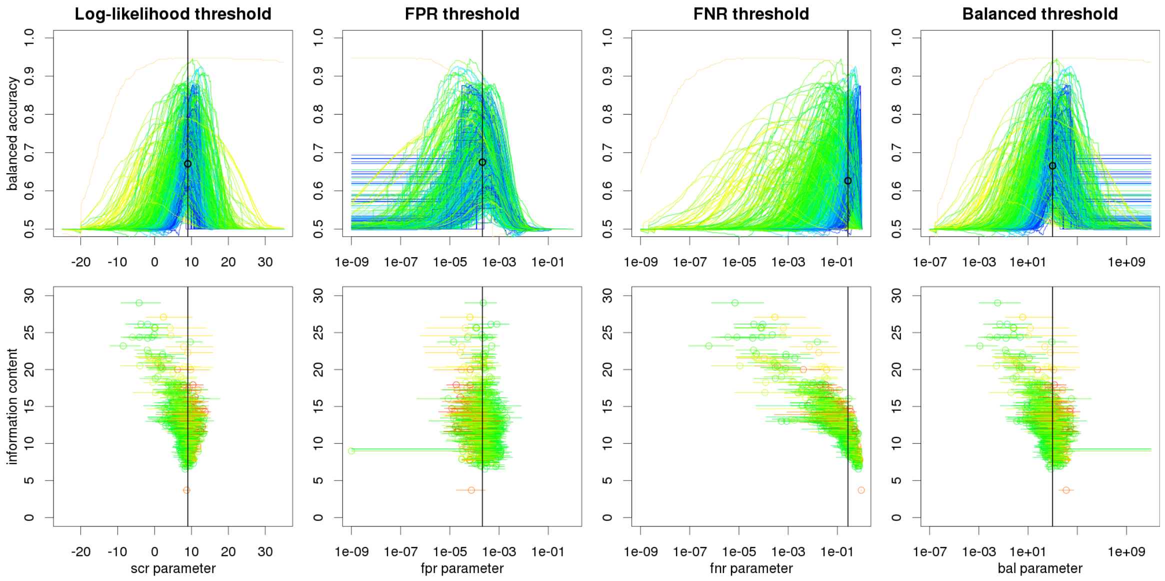

Figure 2: Balanced accuracies for various approaches to threshold selection (vs shuffled peaks). Top row: balanced accuracy vs threshold parameter. Colors represent motif information content: from blue (low), through green and yellow to beige (high). Vertical black lines indicate optimal thresholds, black circles indicate corresponding average balanced accuracies. Bottom row shows how (sub-)optimal parameter values of a motif (X-axis) depends on its information content. For each motif, a circle represents parameter value yielding maximal balanced accuracy and a horizontal line represents a parameter range, for which BA is at least 95% of the maximum. Colors represent motif AUC: from green (low), through yellow to red (high). Balanced accuracies are calculated with respect to negative sequences composed of ChIP-seq peaks shuffled with BiasAway.

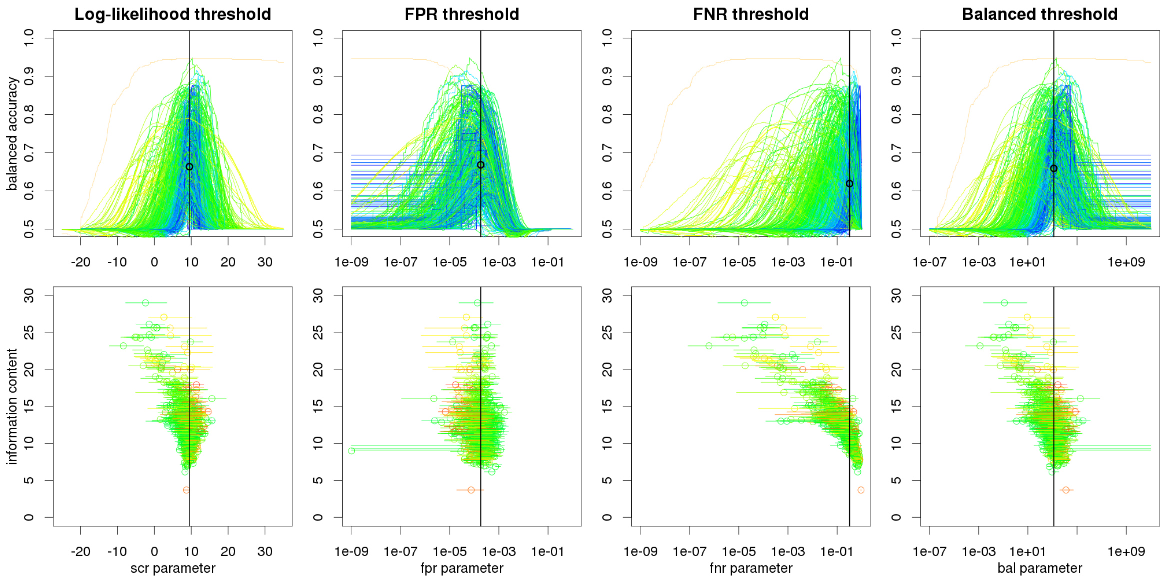

Figure 3: Balanced accuracies for various approaches to threshold selection (vs 2nd order Markov chain). Top row: balanced accuracy vs threshold parameter. Colors represent motif information content: from blue (low), through green and yellow to beige (high). Vertical black lines indicate optimal thresholds, black circles indicate corresponding average balanced accuracies. Bottom row shows how (sub-)optimal parameter values of a motif (X-axis) depends on its information content. For each motif, a circle represents parameter value yielding maximal balanced accuracy and a horizontal line represents a parameter range, for which BA is at least 95% of the maximum. Colors represent motif AUC: from green (low), through yellow to red (high). Balanced accuracies are calculated with respect to negative sequences generated by 2nd order Markov chain learned on ChIP-seq peaks.

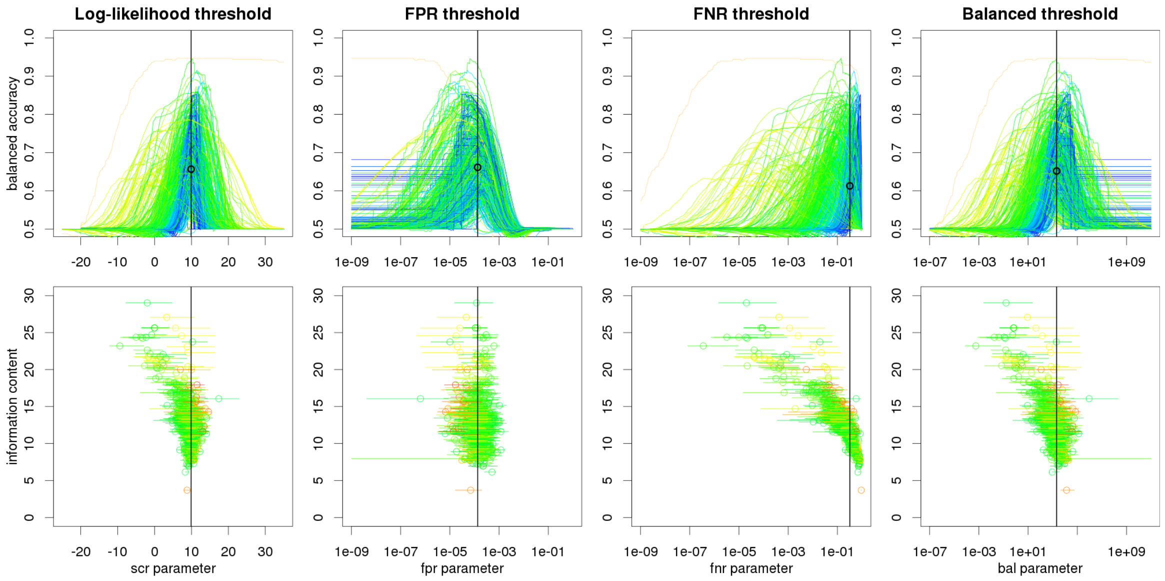

Figure 4: Balanced accuracies for various approaches to threshold selection (vs 3rd order Markov chain). Top row: balanced accuracy vs threshold parameter. Colors represent motif information content: from blue (low), through green and yellow to beige (high). Vertical black lines indicate optimal thresholds, black circles indicate corresponding average balanced accuracies. Bottom row shows how (sub-)optimal parameter values of a motif (X-axis) depends on its information content. For each motif, a circle represents parameter value yielding maximal balanced accuracy and a horizontal line represents a parameter range, for which BA is at least 95% of the maximum. Colors represent motif AUC: from green (low), through yellow to red (high). Balanced accuracies are calculated with respect to negative sequences generated by 3rd order Markov chain learned on ChIP-seq peaks.

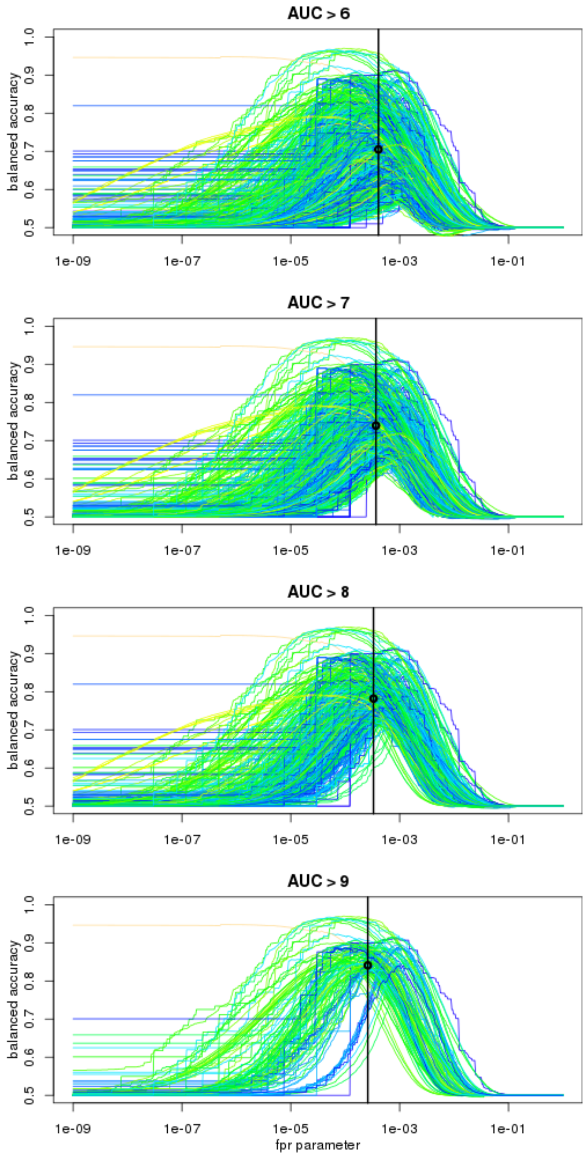

Figure 5: Balanced accuracy versus the FPR threshold for various AUC cutoffs (vs 3rd exons). Colors etc. as on Figures 1 and 2, top row. Balanced accuracies are calculated with respect to negative sequences composed of 3rd exons.

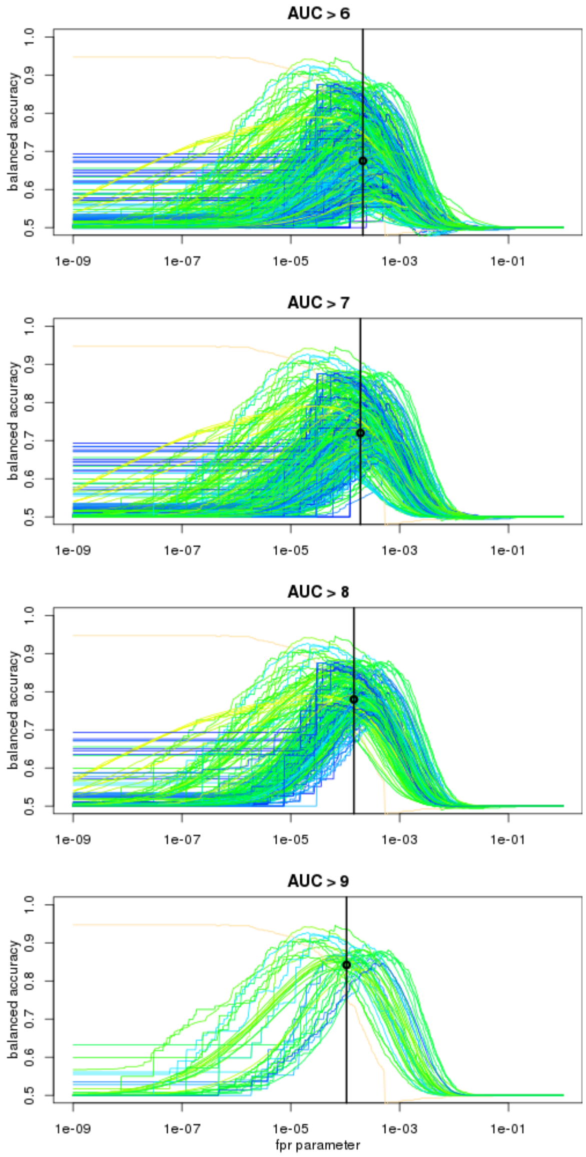

Figure 6: Balanced accuracy versus the FPR threshold for various AUC cutoffs (vs shuffled peaks). Colors etc. as on Figures 1 and 2, top row. Balanced accuracies are calculated with respect to negative sequences composed of ChIP-seq peaks shuffled with BiasAway.

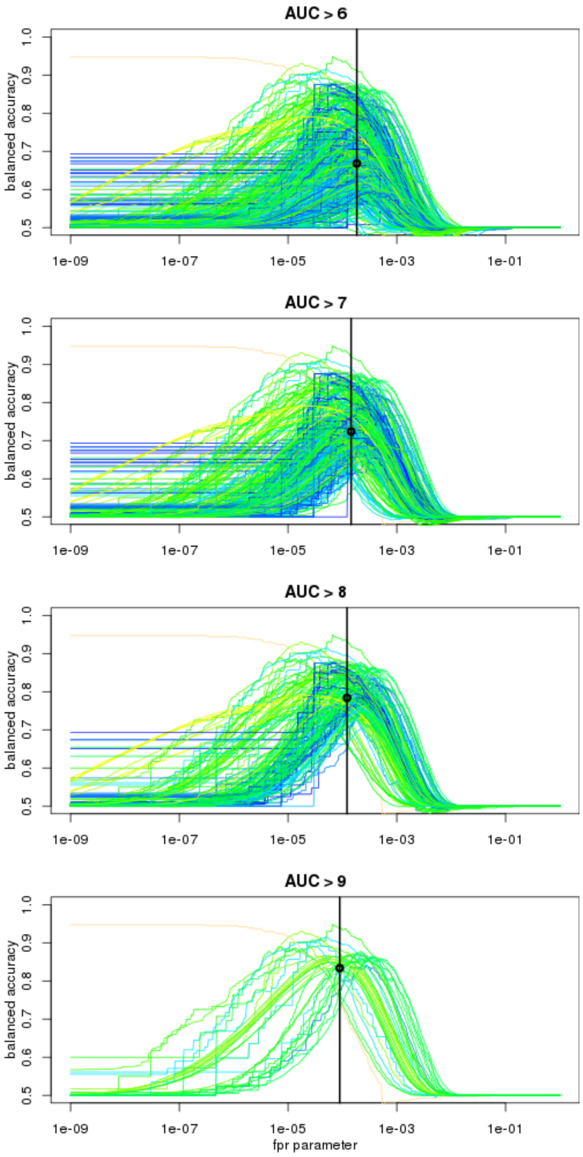

Figure 7: Balanced accuracy versus the FPR threshold for various AUC cutoffs (vs 2nd order Markov chain). Colors etc. as on Figures 1 and 2, top row. Balanced accuracies are calculated with respect to negative sequences generated by 2nd order Markov chain learned on ChIP-seq peaks.

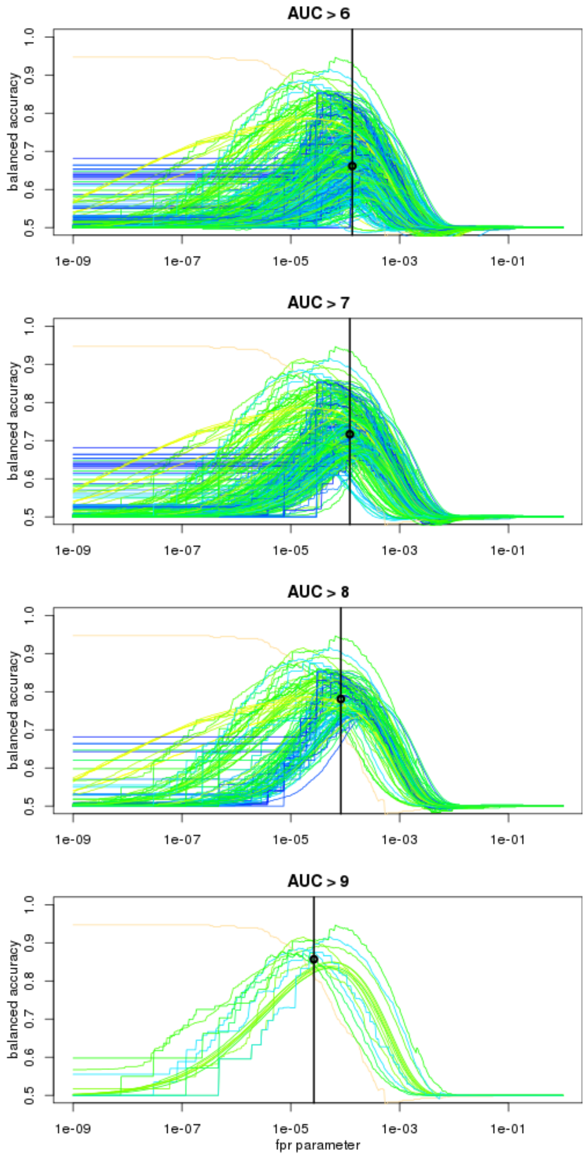

Figure 8: Balanced accuracy versus the FPR threshold for various AUC cutoffs (vs 3rd order Markov chain). Colors etc. as on Figures 1 and 2, top row. Balanced accuracies are calculated with respect to negative sequences generated by 3rd order Markov chain learned on ChIP-seq peaks.

Table 1: Optimal motifs for negative dataset **3rd exons**

ENCODE/funcgen	MatBase		Transfac Prof.		Jaspar vert.		Hocomoco		HT-Selex		SwissRegulon	
TF name	AUC	matrix name	AUC	matrix name	AUC	matrix name	AUC	matrix name	AUC	matrix name	AUC	matrix name
ATF3	**0.764**	**V$CREB.02**	0.705	M00981	–	–	0.694	M00015	–	–	–	–
Ap2alpha	**0.888**	**V$AP2.02**	0.886	M01045	0.883	MA0003.2	0.868	M00004	0.861	selex292	0.856	TFAP2A,C.p2
Ap2gamma	**0.885**	**V$AP2.02**	0.864	M00470	0.866	MA0524.1	0.863	M00006	0.864	selex298	0.859	TFAP2A,C.p2
BHLHE40	0.950	V$BHLHB2.01	0.809	M01034	0.887	MA0464.1	0.956	M00022	**0.969**	**selex316**	0.917	ARNT_ARNT2_BHLHB2_ MAX_MYC_USF1.p2
CTCF	0.929	V$CTCF.04	0.931	M01259	**0.942**	**MA0139.1**	0.940	M00045	0.922	selex2	0.934	CTCF.p2
Cfos	0.777	V$AP1.01	0.786	M00517	0.772	MA0476.1	**0.790**	**M00093**	–	–	0.777	FOS_FOSB,L1_JUNB,D.p2
Cjun	0.851	V$AP1.01	0.854	M00925	0.843	MA0099.1	**0.862**	**M00183**	–	–	0.628	JUN.p2
Cmyc	0.812	V$MYCMAX.03	**0.813**	**M00322**	0.784	MA0147.1	0.772	M00216	–	–	0.745	ARNT_ARNT2_BHLHB2_ MAX_MYC_USF1.p2
E2F1	**0.968**	**V$E2F2.01**	0.963	M00803	0.937	MA0024.2	0.893	M00052	0.921	selex750	0.866	E2F1..5.p2
E2F4	**0.850**	**V$E2F4.01**	0.846	M00803	0.844	MA0470.1	0.786	M00055	0.601	selex753	0.795	E2F1..5.p2
E2F6	**0.891**	**V$E2F4.01**	0.498	M01252	0.810	MA0471.1	0.853	M00057	–	–	–	–
EBF	0.782	V$EBF1.01	0.782	M01871	0.770	MA0154.1	0.782	M00037	**0.792**	**selex79**	0.740	EBF1.p2
ELF1	**0.907**	**V$ELF1.01**	0.882	M02053	0.839	MA0473.1	0.868	M00065	0.876	selex81	0.842	ELF1,2,4.p2
ETS1	0.823	V$ELK3.01	0.804	M02063	0.766	MA0098.1	0.739	M00082	**0.823**	**selex100**	0.739	ETS1,2.p2
Egr1	0.936	V$EGR1.03	0.947	M01873	0.936	PB0010.1	**0.955**	**M00060**	0.949	selex4	0.946	EGR1..3.p2
FOSL1	0.903	V$AP1.02	**0.911**	**M00517**	0.908	MA0477.1	0.891	M00091	–	–	0.902	FOS_FOSB,L1_JUNB,D.p2
FOSL2	0.899	V$AP1.01	0.892	M00925	0.901	MA0478.1	**0.908**	**M00092**	–	–	0.879	FOSL2.p2
FOXA1	0.845	V$HNF3B.03	0.877	M01261	**0.886**	**MA0148.3**	0.867	M00094	–	–	–	–
FOXA2	0.834	V$HNF3B.03	0.822	M02014	**0.886**	**MA0047.2**	0.878	M00095	–	–	**0.886**	**FOXA2.p3**
Gabp	0.908	V$ELK1.03	0.913	M02074	**0.921**	**MA0062.2**	0.915	M00116	0.913	selex116	0.911	ELK1,4_GABPA,B1.p3
Gata1	**0.711**	**V$GATA5.01**	0.685	M00203	0.683	MA0035.3	0.697	M00117	–	–	0.479	GATA1..3.p2
Gata2	**0.902**	**V$GATA2.03**	0.892	M00789	0.890	MA0036.2	0.894	M00118	–	–	0.609	GATA1..3.p2
HNF4A	0.822	V$HNF4.01	0.859	M02220	0.864	MA0114.2	**0.866**	**M00147**	0.859	selex671	0.831	HNF4A_NR2F1,2.p2
HNF4G	0.881	V$HNF4.01	0.831	M00764	**0.912**	**MA0484.1**	0.828	M00148	–	–	–	–
IRF4	**0.755**	**V$IRF1.01**	0.725	M00772	0.743	PB0034.1	0.703	M00174	0.745	selex148	–	–
Junb	0.940	V$AP1.01	0.937	M00925	**0.943**	**MA0490.1**	0.940	M00181	–	–	0.934	FOS_FOSB,L1_JUNB,D.p2
Jund	0.855	V$AP1.01	0.854	M00925	0.854	MA0491.1	**0.861**	**M00182**	–	–	0.849	FOS_FOSB,L1_JUNB,D.p2
MEF2A	0.790	V$MEF2.02	0.794	M00231	**0.800**	**MA0052.2**	0.777	M00204	0.778	selex156	0.769	MEF2A,B,C,D.p2
MEF2C	0.839	V$MEF2.02	0.799	M00941	**0.849**	**MA0497.1**	0.815	M00205	–	–	0.813	MEF2A,B,C,D.p2
Max	0.822	V$MYCMAX.03	**0.824**	**M00322**	0.766	PB0043.1	0.779	M00199	0.787	selex326	0.782	ARNT_ARNT2_BHLHB2_ MAX_MYC_USF1.p2
NFKB	**0.926**	**V$NFKAPPAB65.02**	0.916	M00774	0.905	MA0105.3	0.904	M00235	0.832	selex189	0.892	NFKB1_REL_RELA.p2
NR4A1	**0.793**	**V$NBRE.01**	0.787	M01217	–	–	0.785	M00259	–	–	–	–
Nanog	0.661	V$HOXA2.01	**0.726**	**M01247**	–	–	0.654	M00221	–	–	0.653	NANOGmouse.p2
Nfe2	0.871	V$NFE2.01	0.862	M00037	0.890	MA0501.1	**0.893**	**M00231**	0.802	selex392	0.858	NFE2.p2
Nrf1	0.992	V$NRF1.01	0.994	M02102	0.990	MA0506.1	0.994	M00264	**0.995**	**selex194**	0.994	NRF1.p2
Nrsf	0.858	V$NRSF.02	**0.891**	**M01256**	0.864	MA0138.2	0.866	M00316	–	–	0.861	REST.p3
POU2F2	**0.531**	**V$OCT1.02**	0.508	M00210	0.502	MA0507.1	0.519	M00290	0.503	selex209	0.503	POU2F1..3.p2
POU5F1	0.898	V$OCT3_4.02	0.895	M01125	**0.910**	**MA0142.1**	0.910	M00294	–	–	0.895	POU5F1_SOX2dimer.p2
PU1	**0.932**	**V$SPI1.05**	0.888	M01203	0.914	MA0080.3	0.925	M00350	0.870	selex123	0.884	SPI1.p2
Pax5	0.660	V$PAX5.01	0.660	M00143	0.801	MA0014.2	0.787	M00274	**0.814**	**selex200**	0.654	PAX5.p2
Pbx3	0.739	V$PBX1_MEIS1.01	0.558	M00998	–	–	**0.758**	**M00280**	–	–	–	–
RXRA	0.746	V$PPAR_RXR.02	0.668	M00965	0.761	MA0512.1	0.741	M00326	**0.768**	**selex710**	0.759	RXRG_dimer.p3
SP1	0.606	V$GC.01	0.600	M00932	**0.614**	**MA0079.3**	0.602	M00345	0.597	selex29	0.602	SP1.p2
SP2	**0.903**	**V$SP1.02**	0.896	M01783	0.897	MA0516.1	0.867	M00347	–	–	–	–
Srf	0.694	V$SRF.05	**0.720**	**M00186**	0.672	MA0083.1	0.659	M00355	0.667	selex159	0.678	SRF.p3
Tcf12	**0.742**	**V$ASCL2.01**	0.708	M00698	0.727	MA0521.1	0.721	M00152	–	–	0.591	TAL1_TCF3,4,12.p2
Tr4	0.672	V$COUP.01	0.700	M01776	**0.744**	**MA0504.1**	0.672	M00256	0.681	selex676	–	–
USF1	**0.960**	**V$USF1.02**	0.947	M00121	0.914	MA0093.2	0.951	M00396	0.942	selex352	0.943	ARNT_ARNT2_BHLHB2_ MAX_MYC_USF1.p2
Yy1	**0.839**	**V$YY1.03**	0.799	M02044	0.759	MA0095.2	0.778	M00394	0.807	selex33	0.680	YY1.p2
ZBTB33	0.533	V$KAISO.01	0.561	M01119	**0.931**	**MA0527.1**	0.802	M00184	–	–	–	–
ZBTB7A	**0.891**	**V$ZF9.01**	0.855	M01100	–	–	0.818	M00404	0.824	selex37	–	–
ZEB1	0.811	V$ZEB1.01	0.780	M00414	0.777	MA0103.2	0.750	M00409	–	–	**0.830**	**ZEB1.p2**
Znf263	0.844	V$ZNF263.01	**0.884**	**M01587**	0.787	MA0528.1	–	–	–	–	–	–

Table 2: Optimal motifs for negative dataset **Shuffled peaks**

ENCODE/funcgen	MatBase		Transfac Prof.		Jaspar vert.		Hocomoco		HT-Selex		SwissRegulon	
TF name	AUC	matrix name	AUC	matrix name	AUC	matrix name	AUC	matrix name	AUC	matrix name	AUC	matrix name
ATF3	**0.764**	**V$CREB.02**	0.705	M00981	–	–	0.703	M00015	–	–	–	–
Ap2alpha	**0.888**	**V$AP2.02**	0.886	M01045	0.883	MA0003.2	0.868	M00004	0.861	selex292	0.856	TFAP2A,C.p2
Ap2gamma	**0.885**	**V$AP2.02**	0.864	M00470	0.866	MA0524.1	0.863	M00006	0.864	selex298	0.859	TFAP2A,C.p2
BHLHE40	0.950	V$BHLHB2.01	0.809	M01034	0.893	MA0464.1	0.956	M00022	**0.969**	**selex316**	0.917	ARNT_ARNT2_BHLHB2_ MAX_MYC_USF1.p2
CTCF	0.929	V$CTCF.04	0.931	M01259	**0.942**	**MA0139.1**	0.940	M00045	0.922	selex2	0.934	CTCF.p2
Cfos	0.777	V$AP1.01	0.786	M00517	0.772	MA0476.1	**0.790**	**M00093**	–	–	0.777	FOS_FOSB,L1_JUNB,D.p2
Cjun	0.851	V$AP1.01	0.854	M00925	0.843	MA0099.1	**0.862**	**M00183**	–	–	0.628	JUN.p2
Cmyc	0.812	V$MYCMAX.03	**0.813**	**M00322**	0.784	MA0147.1	0.772	M00216	–	–	0.745	ARNT_ARNT2_BHLHB2_ MAX_MYC_USF1.p2
E2F1	**0.968**	**V$E2F2.01**	0.963	M00803	0.937	MA0024.2	0.893	M00052	0.921	selex750	0.866	E2F1..5.p2
E2F4	**0.850**	**V$E2F4.01**	0.846	M00803	0.844	MA0470.1	0.786	M00055	0.601	selex753	0.795	E2F1..5.p2
E2F6	**0.891**	**V$E2F4.01**	0.576	M01252	0.810	MA0471.1	0.853	M00057	–	–	–	–
EBF	0.782	V$EBF1.01	0.782	M01871	0.770	MA0154.1	0.782	M00037	**0.792**	**selex79**	0.740	EBF1.p2
ELF1	**0.907**	**V$ELF1.01**	0.882	M02053	0.839	MA0473.1	0.868	M00065	0.876	selex81	0.842	ELF1,2,4.p2
ETS1	0.823	V$ELK3.01	0.804	M02063	0.766	MA0098.1	0.739	M00082	**0.823**	**selex100**	0.739	ETS1,2.p2
Egr1	0.936	V$EGR1.03	0.947	M01873	0.936	PB0010.1	**0.955**	**M00060**	0.949	selex4	0.946	EGR1..3.p2
FOSL1	0.903	V$AP1.02	**0.911**	**M00517**	0.908	MA0477.1	0.891	M00091	–	–	0.902	FOS_FOSB,L1_JUNB,D.p2
FOSL2	0.899	V$AP1.01	0.892	M00925	0.901	MA0478.1	**0.908**	**M00092**	–	–	0.879	FOSL2.p2
FOXA1	0.845	V$HNF3B.03	0.877	M01261	**0.886**	**MA0148.3**	0.867	M00094	–	–	–	–
FOXA2	0.834	V$HNF3B.03	0.822	M02014	**0.886**	**MA0047.2**	0.878	M00095	–	–	**0.886**	**FOXA2.p3**
Gabp	0.908	V$ELK1.03	0.913	M02074	**0.921**	**MA0062.2**	0.915	M00116	0.913	selex116	0.911	ELK1,4_GABPA,B1.p3
Gata1	**0.711**	**V$GATA5.01**	0.685	M00203	0.683	MA0035.3	0.697	M00117	–	–	0.604	GATA1..3.p2
Gata2	**0.902**	**V$GATA2.03**	0.892	M00789	0.890	MA0036.2	0.894	M00118	–	–	0.609	GATA1..3.p2
HNF4A	0.822	V$HNF4.01	0.859	M02220	0.864	MA0114.2	**0.866**	**M00147**	0.859	selex671	0.831	HNF4A_NR2F1,2.p2
HNF4G	0.881	V$HNF4.01	0.831	M00764	**0.912**	**MA0484.1**	0.828	M00148	–	–	–	–
IRF4	0.755	V$IRF1.01	0.731	M00772	0.743	PB0034.1	**0.775**	**M00174**	0.745	selex148	–	–
Junb	0.940	V$AP1.01	0.937	M00925	**0.943**	**MA0490.1**	0.940	M00181	–	–	0.934	FOS_FOSB,L1_JUNB,D.p2
Jund	0.855	V$AP1.01	0.854	M00925	0.854	MA0491.1	**0.861**	**M00182**	–	–	0.849	FOS_FOSB,L1_JUNB,D.p2
MEF2A	0.790	V$MEF2.02	0.794	M00231	**0.800**	**MA0052.2**	0.777	M00204	0.778	selex156	0.769	MEF2A,B,C,D.p2
MEF2C	0.839	V$MEF2.02	0.799	M00941	**0.849**	**MA0497.1**	0.815	M00205	–	–	0.813	MEF2A,B,C,D.p2
Max	0.822	V$MYCMAX.03	**0.824**	**M00322**	0.766	PB0043.1	0.779	M00199	0.787	selex326	0.782	ARNT_ARNT2_BHLHB2_ MAX_MYC_USF1.p2
NFKB	**0.926**	**V$NFKAPPAB65.02**	0.916	M00774	0.905	MA0105.3	0.904	M00235	0.832	selex189	0.892	NFKB1_REL_RELA.p2
NR4A1	**0.793**	**V$NBRE.01**	0.787	M01217	–	–	0.785	M00259	–	–	–	–
Nanog	0.661	V$HOXA2.01	**0.771**	**M01247**	–	–	0.654	M00221	–	–	0.653	NANOGmouse.p2
Nfe2	0.871	V$NFE2.01	0.862	M00037	0.890	MA0501.1	**0.893**	**M00231**	0.802	selex392	0.858	NFE2.p2
Nrf1	0.992	V$NRF1.01	0.994	M02102	0.990	MA0506.1	0.994	M00264	**0.995**	**selex194**	0.994	NRF1.p2
Nrsf	0.858	V$NRSF.02	**0.891**	**M01256**	0.864	MA0138.2	0.866	M00316	–	–	0.861	REST.p3
POU2F2	0.587	V$OCT2.01	0.596	M03836	0.596	MA0507.1	0.590	M00290	**0.597**	**selex209**	0.571	POU2F1..3.p2
POU5F1	0.898	V$OCT3_4.02	0.895	M01125	**0.910**	**MA0142.1**	0.910	M00294	–	–	0.895	POU5F1_SOX2dimer.p2
PU1	0.940	V$SPI1.05	0.910	M01172	0.926	MA0080.3	**0.942**	**M00350**	0.870	selex123	0.890	SPI1.p2
Pax5	0.660	V$PAX5.01	0.660	M00143	0.801	MA0014.2	0.787	M00274	**0.814**	**selex200**	0.654	PAX5.p2
Pbx3	0.739	V$PBX1_MEIS1.01	0.558	M00998	–	–	**0.758**	**M00280**	–	–	–	–
RXRA	0.746	V$PPAR_RXR.02	0.668	M00965	0.761	MA0512.1	0.741	M00326	**0.768**	**selex710**	0.759	RXRG_dimer.p3
SP1	0.606	V$GC.01	0.604	M02281	**0.628**	**MA0079.3**	0.623	M00346	0.597	selex29	0.609	SP1.p2
SP2	**0.903**	**V$SP1.02**	0.896	M01783	0.897	MA0516.1	0.867	M00347	–	–	–	–
Srf	0.694	V$SRF.05	**0.720**	**M00186**	0.679	MA0083.2	0.672	M00355	0.675	selex160	0.678	SRF.p3
Tcf12	**0.742**	**V$ASCL2.01**	0.708	M00698	0.727	MA0521.1	0.721	M00152	–	–	0.591	TAL1_TCF3,4,12.p2
Tr4	0.672	V$COUP.01	0.700	M01776	**0.744**	**MA0504.1**	0.672	M00256	0.681	selex676	–	–
USF1	**0.960**	**V$USF1.02**	0.947	M00121	0.914	MA0093.2	0.951	M00396	0.942	selex352	0.943	ARNT_ARNT2_BHLHB2_ MAX_MYC_USF1.p2
Yy1	**0.839**	**V$YY1.03**	0.799	M02044	0.772	MA0095.2	0.784	M00394	0.807	selex33	0.680	YY1.p2
ZBTB33	0.533	V$KAISO.01	0.561	M01119	**0.931**	**MA0527.1**	0.802	M00184	–	–	–	–
ZBTB7A	**0.891**	**V$ZF9.01**	0.855	M01100	–	–	0.818	M00404	0.824	selex37	–	–
ZEB1	0.811	V$ZEB1.01	0.780	M00414	0.777	MA0103.2	0.750	M00409	–	–	**0.830**	**ZEB1.p2**
Znf263	0.844	V$ZNF263.01	**0.884**	**M01587**	0.787	MA0528.1	–	–	–	–	–	–

Table 3: Optimal motifs for negative dataset **2nd order Markov chain**

ENCODE/funcgen	MatBase		Transfac Prof.		Jaspar vert.		Hocomoco		HT-Selex		SwissRegulon	
TF name	AUC	matrix name	AUC	matrix name	AUC	matrix name	AUC	matrix name	AUC	matrix name	AUC	matrix name
ATF3	**0.764**	**V$CREB.02**	0.705	M00981	–	–	0.703	M00015	–	–	–	–
Ap2alpha	0.888	V$AP2.02	0.886	M01045	**0.892**	**MA0003.2**	0.868	M00004	0.861	selex292	0.856	TFAP2A,C.p2
Ap2gamma	0.885	V$AP2.02	**0.890**	**M01859**	0.882	MA0524.1	0.863	M00006	0.864	selex298	0.859	TFAP2A,C.p2
BHLHE40	0.950	V$BHLHB2.01	0.822	M01034	0.893	MA0464.1	0.956	M00022	**0.969**	**selex316**	0.917	ARNT_ARNT2_BHLHB2_ MAX_MYC_USF1.p2
CTCF	0.943	V$CTCF.05	0.951	M01259	**0.956**	**MA0139.1**	0.951	M00045	0.922	selex2	0.954	CTCF.p2
Cfos	0.777	V$AP1.01	0.786	M00517	0.772	MA0476.1	**0.790**	**M00093**	–	–	0.777	FOS_FOSB,L1_JUNB,D.p2
Cjun	0.851	V$AP1.01	0.854	M00925	0.843	MA0099.1	**0.862**	**M00183**	–	–	0.628	JUN.p2
Cmyc	0.812	V$MYCMAX.03	**0.813**	**M00322**	0.784	MA0147.1	0.772	M00216	–	–	0.745	ARNT_ARNT2_BHLHB2_ MAX_MYC_USF1.p2
E2F1	**0.968**	**V$E2F2.01**	0.963	M00803	0.937	MA0024.2	0.893	M00052	0.921	selex750	0.866	E2F1..5.p2
E2F4	**0.850**	**V$E2F4.01**	0.846	M00803	0.844	MA0470.1	0.786	M00055	0.601	selex753	0.795	E2F1..5.p2
E2F6	**0.891**	**V$E2F4.01**	0.576	M01252	0.810	MA0471.1	0.853	M00057	–	–	–	–
EBF	0.782	V$EBF1.01	0.782	M01871	0.787	MA0154.2	0.782	M00037	**0.792**	**selex79**	0.740	EBF1.p2
ELF1	**0.907**	**V$ELF1.01**	0.882	M02053	0.839	MA0473.1	0.868	M00065	0.876	selex81	0.842	ELF1,2,4.p2
ETS1	0.823	V$ELK3.01	0.804	M02063	0.766	MA0098.1	0.742	M00082	**0.823**	**selex100**	0.739	ETS1,2.p2
Egr1	0.936	V$EGR1.03	0.947	M01873	0.936	PB0010.1	**0.955**	**M00060**	0.949	selex4	0.946	EGR1..3.p2
FOSL1	0.903	V$AP1.02	**0.911**	**M00517**	0.908	MA0477.1	0.891	M00091	–	–	0.902	FOS_FOSB,L1_JUNB,D.p2
FOSL2	0.899	V$AP1.01	0.892	M00925	0.901	MA0478.1	**0.908**	**M00092**	–	–	0.879	FOSL2.p2
FOXA1	0.845	V$HNF3B.03	0.877	M01261	**0.886**	**MA0148.3**	0.867	M00094	–	–	–	–
FOXA2	0.834	V$HNF3B.03	0.822	M02014	**0.886**	**MA0047.2**	0.878	M00095	–	–	**0.886**	**FOXA2.p3**
Gabp	0.908	V$ELK1.03	0.913	M02074	**0.921**	**MA0062.2**	0.915	M00116	0.913	selex116	0.911	ELK1,4_GABPA,B1.p3
Gata1	**0.711**	**V$GATA5.01**	0.685	M00203	0.683	MA0035.3	0.697	M00117	–	–	0.625	GATA1..3.p2
Gata2	**0.902**	**V$GATA2.03**	0.892	M00789	0.890	MA0036.2	0.894	M00118	–	–	0.654	GATA1..3.p2
HNF4A	0.822	V$HNF4.01	0.859	M02220	0.865	MA0114.2	**0.866**	**M00147**	0.859	selex671	0.831	HNF4A_NR2F1,2.p2
HNF4G	0.881	V$HNF4.01	0.831	M00764	**0.920**	**MA0484.1**	0.861	M00148	–	–	–	–
IRF4	0.755	V$IRF1.01	0.731	M00772	0.743	PB0034.1	**0.775**	**M00174**	0.745	selex148	–	–
Junb	0.940	V$AP1.01	0.937	M00925	**0.943**	**MA0490.1**	0.940	M00181	–	–	0.934	FOS_FOSB,L1_JUNB,D.p2
Jund	0.855	V$AP1.01	0.854	M00925	0.854	MA0491.1	**0.861**	**M00182**	–	–	0.849	FOS_FOSB,L1_JUNB,D.p2
MEF2A	0.790	V$MEF2.02	0.794	M00231	**0.800**	**MA0052.2**	0.777	M00204	0.778	selex156	0.769	MEF2A,B,C,D.p2
MEF2C	0.839	V$MEF2.02	0.799	M00941	**0.849**	**MA0497.1**	0.815	M00205	–	–	0.813	MEF2A,B,C,D.p2
Max	0.822	V$MYCMAX.03	**0.824**	**M00322**	0.766	PB0043.1	0.779	M00199	0.787	selex326	0.782	ARNT_ARNT2_BHLHB2_ MAX_MYC_USF1.p2
NFKB	**0.926**	**V$NFKAPPAB65.02**	0.916	M00774	0.905	MA0105.3	0.904	M00235	0.832	selex189	0.892	NFKB1_REL_RELA.p2
NR4A1	**0.793**	**V$NBRE.01**	0.787	M01217	–	–	0.785	M00259	–	–	–	–
Nanog	0.661	V$HOXA2.01	**0.772**	**M01247**	–	–	0.654	M00221	–	–	0.653	NANOGmouse.p2
Nfe2	**0.935**	**V$NRL.02**	0.870	M02104	0.897	MA0501.1	0.896	M00231	0.802	selex392	0.858	NFE2.p2
Nrf1	0.992	V$NRF1.01	0.994	M02102	0.990	MA0506.1	0.994	M00264	**0.995**	**selex194**	0.994	NRF1.p2
Nrsf	0.863	V$NRSF.01	**0.891**	**M01256**	0.864	MA0138.2	0.866	M00316	–	–	0.861	REST.p3
POU2F2	**0.660**	**V$OCT1.02**	0.630	M03836	0.604	MA0507.1	0.620	M00290	0.620	selex232	0.621	POU2F1..3.p2
POU5F1	**0.931**	**V$OCT3_4.02**	0.895	M01125	0.914	MA0142.1	0.917	M00294	–	–	0.914	POU5F1.p2
PU1	0.940	V$SPI1.05	0.910	M01172	0.926	MA0080.3	**0.942**	**M00350**	0.870	selex123	0.890	SPI1.p2
Pax5	0.660	V$PAX5.01	0.664	M03577	0.801	MA0014.2	0.787	M00274	**0.814**	**selex200**	0.654	PAX5.p2
Pbx3	0.750	V$PBX1_MEIS1.01	0.558	M00998	–	–	**0.764**	**M00280**	–	–	–	–
RXRA	0.746	V$PPAR_RXR.02	0.668	M00965	**0.794**	**MA0512.1**	0.741	M00326	0.768	selex710	0.759	RXRG_dimer.p3
SP1	0.606	V$GC.01	0.610	M00008	**0.628**	**MA0079.3**	0.623	M00346	0.597	selex29	0.609	SP1.p2
SP2	**0.903**	**V$SP1.02**	0.896	M01783	0.897	MA0516.1	0.867	M00347	–	–	–	–
Srf	0.729	V$SRF.03	**0.734**	**M00215**	0.710	MA0083.2	0.723	M00355	0.684	selex159	0.703	SRF.p3
Tcf12	**0.916**	**V$ASCL2.01**	0.878	M00698	0.892	MA0521.1	0.892	M00152	–	–	0.724	TAL1_TCF3,4,12.p2
Tr4	0.672	V$COUP.01	0.714	M01776	**0.744**	**MA0504.1**	0.672	M00256	0.681	selex676	–	–
USF1	**0.960**	**V$USF1.02**	0.947	M00121	0.932	MA0093.2	0.951	M00396	0.947	selex352	0.943	ARNT_ARNT2_BHLHB2_ MAX_MYC_USF1.p2
Yy1	0.839	V$YY1.03	0.833	M01035	0.821	MA0095.2	**0.841**	**M00394**	0.823	selex33	0.758	YY1.p2
ZBTB33	0.655	V$KAISO.01	0.671	M01119	**0.931**	**MA0527.1**	0.802	M00184	–	–	–	–
ZBTB7A	**0.891**	**V$ZF9.01**	0.855	M01100	–	–	0.818	M00404	0.824	selex37	–	–
ZEB1	**0.866**	**V$ZEB1.01**	0.788	M00412	0.843	MA0103.2	0.816	M00409	–	–	0.830	ZEB1.p2
Znf263	0.844	V$ZNF263.01	**0.884**	**M01587**	0.787	MA0528.1	–	–	–	–	–	–

Table 4: Optimal motifs for negative dataset **3rd order Markov chain**

ENCODE/funcgen	MatBase		Transfac Prof.		Jaspar vert.		Hocomoco		HT-Selex		SwissRegulon	
TF name	AUC	matrix name	AUC	matrix name	AUC	matrix name	AUC	matrix name	AUC	matrix name	AUC	matrix name
ATF3	**0.764**	**V$CREB.02**	0.705	M00981	–	–	0.703	M00015	–	–	–	–
Ap2alpha	0.888	V$AP2.02	0.886	M01045	**0.892**	**MA0003.2**	0.868	M00004	0.861	selex292	0.856	TFAP2A,C.p2
Ap2gamma	0.885	V$AP2.02	**0.890**	**M01859**	0.882	MA0524.1	0.863	M00006	0.864	selex298	0.859	TFAP2A,C.p2
BHLHE40	0.950	V$BHLHB2.01	0.822	M01034	0.893	MA0464.1	0.956	M00022	**0.969**	**selex316**	0.917	ARNT_ARNT2_BHLHB2_MAX_MYC_USF1.p2
CTCF	0.943	V$CTCF.05	0.951	M01259	**0.956**	**MA0139.1**	0.951	M00045	0.922	selex2	0.954	CTCF.p2
Cfos	0.777	V$AP1.01	0.786	M00517	0.772	MA0476.1	**0.790**	**M00093**	–	–	0.777	FOS_FOSB,L1_JUNB,D.p2
Cjun	0.851	V$AP1.01	0.854	M00925	0.843	MA0099.1	**0.862**	**M00183**	–	–	0.628	JUN.p2
Cmyc	0.812	V$MYCMAX.03	**0.813**	**M00322**	0.784	MA0147.1	0.772	M00216	–	–	0.745	ARNT_ARNT2_BHLHB2_MAX_MYC_USF1.p2
E2F1	**0.968**	**V$E2F2.01**	0.963	M00803	0.937	MA0024.2	0.893	M00052	0.921	selex750	0.866	E2F1..5.p2
E2F4	**0.850**	**V$E2F4.01**	0.846	M00803	0.844	MA0470.1	0.786	M00055	0.601	selex753	0.795	E2F1..5.p2
E2F6	**0.891**	**V$E2F4.01**	0.576	M01252	0.810	MA0471.1	0.853	M00057	–	–	–	–
EBF	0.782	V$EBF1.01	0.782	M01871	0.787	MA0154.2	0.782	M00037	**0.792**	**selex79**	0.740	EBF1.p2
ELF1	**0.907**	**V$ELF1.01**	0.882	M02053	0.839	MA0473.1	0.868	M00065	0.876	selex81	0.842	ELF1,2,4.p2
ETS1	0.823	V$ELK3.01	0.804	M02063	0.766	MA0098.1	0.742	M00082	**0.823**	**selex100**	0.739	ETS1,2.p2
Egr1	0.936	V$EGR1.03	0.947	M01873	0.936	PB0010.1	**0.955**	**M00060**	0.949	selex4	0.946	EGR1..3.p2
FOSL1	0.903	V$AP1.02	**0.911**	**M00517**	0.908	MA0477.1	0.891	M00091	–	–	0.902	FOS_FOSB,L1_JUNB,D.p2
FOSL2	0.899	V$AP1.01	0.892	M00925	0.901	MA0478.1	**0.908**	**M00092**	–	–	0.879	FOSL2.p2
FOXA1	0.845	V$HNF3B.03	0.877	M01261	**0.886**	**MA0148.3**	0.867	M00094	–	–	–	–
FOXA2	0.834	V$HNF3B.03	0.822	M02014	**0.886**	**MA0047.2**	0.878	M00095	–	–	**0.886**	**FOXA2.p3**
Gabp	0.908	V$ELK1.03	0.913	M02074	**0.921**	**MA0062.2**	0.915	M00116	0.913	selex116	0.911	ELK1,4_GABPA,B1.p3
Gata1	**0.711**	**V$GATA5.01**	0.685	M00203	0.683	MA0035.3	0.697	M00117	–	–	0.625	GATA1..3.p2
Gata2	**0.902**	**V$GATA2.03**	0.892	M00789	0.890	MA0036.2	0.894	M00118	–	–	0.654	GATA1..3.p2
HNF4A	0.822	V$HNF4.01	0.859	M02220	0.865	MA0114.2	**0.866**	**M00147**	0.859	selex671	0.831	HNF4A_NR2F1,2.p2
HNF4G	0.881	V$HNF4.01	0.831	M00764	**0.920**	**MA0484.1**	0.861	M00148	–	–	–	–
IRF4	0.755	V$IRF1.01	0.731	M00772	0.743	PB0034.1	**0.775**	**M00174**	0.745	selex148	–	–
Junb	0.940	V$AP1.01	0.937	M00925	**0.943**	**MA0490.1**	0.940	M00181	–	–	0.934	FOS_FOSB,L1_JUNB,D.p2
Jund	0.855	V$AP1.01	0.854	M00925	0.854	MA0491.1	**0.861**	**M00182**	–	–	0.849	FOS_FOSB,L1_JUNB,D.p2
MEF2A	0.790	V$MEF2.02	0.794	M00231	**0.800**	**MA0052.2**	0.777	M00204	0.778	selex156	0.769	MEF2A,B,C,D.p2
MEF2C	0.839	V$MEF2.02	0.799	M00941	**0.849**	**MA0497.1**	0.815	M00205	–	–	0.813	MEF2A,B,C,D.p2
Max	0.822	V$MYCMAX.03	**0.824**	**M00322**	0.766	PB0043.1	0.779	M00199	0.787	selex326	0.782	ARNT_ARNT2_BHLHB2_MAX_MYC_USF1.p2
NFKB	**0.926**	**V$NFKAPPAB65.02**	0.916	M00774	0.905	MA0105.3	0.904	M00235	0.832	selex189	0.892	NFKB1_REL_RELA.p2
NR4A1	**0.793**	**V$NBRE.01**	0.787	M01217	–	–	0.785	M00259	–	–	–	–
Nanog	0.661	V$HOXA2.01	**0.772**	**M01247**	–	–	0.654	M00221	–	–	0.653	NANOGmouse.p2
Nfe2	**0.935**	**V$NRL.02**	0.870	M02104	0.897	MA0501.1	0.896	M00231	0.802	selex392	0.858	NFE2.p2
Nrf1	0.992	V$NRF1.01	0.994	M02102	0.990	MA0506.1	0.994	M00264	**0.995**	**selex194**	0.994	NRF1.p2
Nrsf	0.863	V$NRSF.01	**0.891**	**M01256**	0.864	MA0138.2	0.866	M00316	–	–	0.861	REST.p3
POU2F2	**0.660**	**V$OCT1.02**	0.630	M03836	0.604	MA0507.1	0.620	M00290	0.620	selex232	0.621	POU2F1..3.p2
POU5F1	**0.931**	**V$OCT3_4.02**	0.895	M01125	0.914	MA0142.1	0.917	M00294	–	–	0.914	POU5F1.p2
PU1	0.940	V$SPI1.05	0.910	M01172	0.926	MA0080.3	**0.942**	**M00350**	0.870	selex123	0.890	SPI1.p2
Pax5	0.660	V$PAX5.01	0.664	M03577	0.801	MA0014.2	0.787	M00274	**0.814**	**selex200**	0.654	PAX5.p2
Pbx3	0.750	V$PBX1_MEIS1.01	0.558	M00998	–	–	**0.764**	**M00280**	–	–	–	–
RXRA	0.746	V$PPAR_RXR.02	0.668	M00965	**0.794**	**MA0512.1**	0.741	M00326	0.768	selex710	0.759	RXRG_dimer.p3
SP1	0.606	V$GC.01	0.610	M00008	**0.628**	**MA0079.3**	0.623	M00346	0.597	selex29	0.609	SP1.p2
SP2	**0.903**	**V$SP1.02**	0.896	M01783	0.897	MA0516.1	0.867	M00347	–	–	–	–
Srf	0.729	V$SRF.03	**0.734**	**M00215**	0.710	MA0083.2	0.723	M00355	0.684	selex159	0.703	SRF.p3
Tcf12	**0.916**	**V$ASCL2.01**	0.878	M00698	0.892	MA0521.1	0.892	M00152	–	–	0.724	TAL1_TCF3,4,12.p2
Tr4	0.672	V$COUP.01	0.714	M01776	**0.744**	**MA0504.1**	0.672	M00256	0.681	selex676	–	–
USF1	**0.960**	**V$USF1.02**	0.947	M00121	0.932	MA0093.2	0.951	M00396	0.947	selex352	0.943	ARNT_ARNT2_BHLHB2_MAX_MYC_USF1.p2
Yy1	0.839	V$YY1.03	0.833	M01035	0.821	MA0095.2	**0.841**	**M00394**	0.823	selex33	0.758	YY1.p2
ZBTB33	0.655	V$KAISO.01	0.671	M01119	**0.931**	**MA0527.1**	0.802	M00184	–	–	–	–
ZBTB7A	**0.891**	**V$ZF9.01**	0.855	M01100	–	–	0.818	M00404	0.824	selex37	–	–
ZEB1	**0.866**	**V$ZEB1.01**	0.788	M00412	0.843	MA0103.2	0.816	M00409	–	–	0.830	ZEB1.p2
Znf263	0.844	V$ZNF263.01	**0.884**	**M01587**	0.787	MA0528.1	–	–	–	–	–	–
